# No More Tears: Mining Sequencing Data for Novel *Bt* Cry Toxins with CryProcessor

**DOI:** 10.3390/toxins12030204

**Published:** 2020-03-23

**Authors:** Anton E. Shikov, Yury V. Malovichko, Rostislav K. Skitchenko, Anton A. Nizhnikov, Kirill S. Antonets

**Affiliations:** 1 Laboratory for Proteomics of Supra-Organismal Systems, All-Russia Research Institute for Agricultural Microbiology (ARRIAM), St. Petersburg 196608, Russia; 2 Faculty of Biology, St. Petersburg State University, St. Petersburg 199034, Russia; 3 Computer Technologies Laboratory, ITMO University, St. Petersburg 197101, Russia

**Keywords:** *Bacillus thuringiensis*, *Bt*, Cry toxins, CryProcessor, pathogen, insecticide, biopesticide

## Abstract

*Bacillus thuringiensis* (*Bt*) is a natural pathogen of insects and some other groups of invertebrates that produces three-domain Cry (3d-Cry) toxins, which are highly host-specific pesticidal proteins. These proteins represent the most commonly used bioinsecticides in the world and are used for commercial purposes on the market of insecticides, being convergent with the paradigm of sustainable growth and ecological development. Emerging resistance to known toxins in pests stresses the need to expand the list of known toxins to broaden the horizons of insecticidal approaches. For this purpose, we have elaborated a fast and user-friendly tool called CryProcessor, which allows productive and precise mining of 3d-Cry toxins. The only existing tool for mining Cry toxins, called a BtToxin_scanner, has significant limitations such as limited query size, lack of accuracy and an outdated database. In order to find a proper solution to these problems, we have developed a robust pipeline, capable of precise 3d-Cry toxin mining. The unique feature of the pipeline is the ability to search for Cry toxins sequences directly on assembly graphs, providing an opportunity to analyze raw sequencing data and overcoming the problem of fragmented assemblies. Moreover, CryProcessor is able to predict precisely the domain layout in arbitrary sequences, allowing the retrieval of sequences of definite domains beyond the bounds of a limited number of toxins presented in CryGetter. Our algorithm has shown efficiency in all its work modes and outperformed its analogues on large amounts of data. Here, we describe its main features and provide information on its benchmarking against existing analogues. CryProcessor is a novel, fast, convenient, open source (https://github.com/lab7arriam/cry_processor), platform-independent, and precise instrument with a console version and elaborated web interface (https://lab7.arriam.ru/tools/cry_processor). Its major merits could make it possible to carry out massive screening for novel 3d-Cry toxins and obtain sequences of specific domains for further comprehensive in silico experiments in constructing artificial toxins.

## 1. Introduction

Crop losses caused by insects continue to be one of the major challenges in agriculture. Exploiting chemical insecticides appears to be losing ground due to the potential toxicity for humans, the detrimental impact on the environment, and the increasing pest resistance to existing chemicals [1,2]. Thankfully, bioinsectides could become an eco-friendly yet cost-effective solution. *Bacillus thuringiensis* (*Bt*) produces a broad range of proteinaceous toxins, which are highly specific and could act as an enormously powerful instrument in the arsenal of insecticidal approaches. Exclusive toxicity against target species makes *Bt* toxins applicable in agriculture and may prevent contagious diseases spread by insects, such as dengue and malaria [3]. The most prevalent *Bt* toxin group is Cry (from “crystal”), and the toxins are produced on the transition to the sporulation stage and show specificity predominantly against species of Lepidoptera, Diptera and Coleoptera orders [4].

Among all Cry toxins, three-domain Cry (3d-Cry) toxins represent the largest group. Despite considerable differences in amino acid sequences, these toxins share a conservative structure [5]. Three domains, responsible for their mode of action, are located at the N-terminus of the proteins. Domain I consists of a bundle of seven α-helices and is involved in pore formation and penetration into insect cells [4]. Domain II comprises three antiparallel β-sheets and it takes part in receptor recognition required for further toxin activation [4]. Domain III is a β-sandwich and is essential for receptor binding as well as pore formation [4].

The first commercial usage of *Bt* toxins as an insecticide dates back to 1938 [6]. Since then, not only *Bt* or isolated toxin solutions have been put into practice but also transgenic plants bearing *cry* genes. By 2018, approximately 191 million hectares of biotech crops have been cultivated all over the world [7]. For instance, a 70% reduction in insecticidal chemical capacities has been reported in India after the introduction of *Bt*-cotton, resulting in savings up to US$30 per hectare and an increase in crop yields of more than 80–87% [8]. During the last decades, *Bt*-derived insecticides have occupied more than 50% of the global biopesticide market [9].

Nonetheless, emerging resistance of insects to Cry toxins threatens sustainable implementation of biopesticides, including *Bt* crops [1]. One strategy to overcome this difficulty is to extend the diversity of Cry toxins used in agriculture, by a comprehensive search for new toxins. Another strategy implies designing artificial toxins. The only available application for searching new Cry toxins is BtToxin_scanner [10]. It allows extracting *Bt* toxins from sets of biological sequences but, unfortunately, it has several limitations that prevent it from being used for large-scale analysis. A web version of BtToxin_scanner allows the processing of limited amounts of data at a time (16 Mb). The tool allows the extraction of Cry toxin sequences directly from Illumina reads only in the standalone version, whose algorithm is poorly described. Furthermore, BtToxin_scanner is not capable of predicting domain structures of identified Cry toxins. Another strategy of expanding the diversity of Cry proteins is to construct artificial toxins by a combination of existing domains and parts of toxins. It requires efficient and precise prediction of domain structures of proteins. For now, there is no specific instrument capable of generating domain structures among the sequences of 3d-Cry toxins. The only tool, which provides data for domain boundaries of Cry toxins, is CryGetter [11]. Nonetheless, it provides data only for a limited predefined set of proteins. It means that, to date, the only available approach is simple manual mining of the domains from arbitrary Cry toxin sequences, utilizing the resources of databases such as Pfam and InterPro. These databases could not rapidly provide the layout of the domains in the case of big queries and are also limited by the sensitivity of the models presented in those databases. Developed by us, the CryProcessor tool successfully copes with both strategies, namely searching Cry toxins in sets of data arose from whole-genome sequencing and predicting their domain structure. The tool is suitable for high-throughput screening and provides unique functionality such as robust and precise searches for Cry proteins in fragmented genome assemblies using raw sequencing data.

## 2. Results

We tested our tool, CryProcessor, in all possible modes and compared it with the existing tool BtToxin_scanner [10]. CryProcessor provides two search modes, “find domains” and “domain only”, using a hidden Markov model (HMM) to extract complete toxins with subsequent domain structure prediction or exploiting domain-specific models only, respectively. Furthermore, the tool can be launched with sequences in FASTA format by default or with raw Illumina reads in “PathRacer” mode. The last mode implies genome assembly with SPAdes [12] and ensuing search for toxin sequences in the assembly graph with PathRacer [13]. It provides a unique possibility to overcome the problem of fragmented assembles; even if parts of *cry* genes are located at different contigs, they are combined by PathRacer, thus enabling the extraction of full sequences.

The modes “find domains” and “domains only” were compared to the BtToxin_scanner standalone version using a dataset comprising protein sets from 511 *Bt* genome assemblies from National Center Biotechnology Information (NCBI) Assembly database (the full list of assembly IDs is presented in Table S1). A comparison of assembly-induced modes of the respective tools was performed on reads from a HD-133 shotgun genome sequencing project (Sequence Read Archive (SRA) accession SRX2330733). To test the tool for false positive results, we chose an SRA project SRR9222607 representing *Mycobacterium tuberculosis* genomic reads as a negative control and launched the tool a “PathRacer” mode with it. Finally, we also compared the tool to the BtToxin_scanner online service [10], in the terms of output specificity on five random *Bt* genome projects. All comparison procedures were performed on an ARRIAM server (1008 Gb RAM, 72 threads with 2.30 GHz per CPU) in multi-thread mode utilizing 72 CPU threads in parallel. 

Both “find domains” and “domains only” modes of CryProcessor appear to outperform BtToxin_scanner [10] in runtime and output size as indicated in Table 1. However, the outputs of the two CryProcessor modes differ in both quantity and particular found entries. To confirm that neither of the modes produced false positives, we chose the excessive entries from the “domains only” output and performed a “find domains” run with them. The results matched the “domains only” output properly, indicating that the “find domains” sensitivity may decrease with the increase of the input depth (discrepancy is proportional to the number of entries in the query). This means that the “find domains” mode, although it succeeds in terms of launch time, tends to lose some toxin entries that can be detected via the “domains only” mode. For consistency, we advise the potential users to either use the “domains only” mode or launch the tool in both modes and merge the results by shared terms.

Regarding the assembly-dependent mode, CryProcessor in “PathRacer” mode is not as effective as BtToxin_scanner [10] in runtime, but CryProcessor still transcends its analog with the *Bt* genome assembly (Table 2). Furthermore, CryProcessor does not produce any positive results with a query completely devoid of toxins (Table 3).

The comparison of the “find domains” mode of CryProcessor to the online version of BtToxin_scanner [10] is presented in Tables S2 and S3. In brief, the BtToxin_scanner is inferior to the CryProcessor tool regarding sensitivity and produces an output containing truncated and fractured sequences according to the Pfam database [14]. This is crucial since incomplete sequences do not provide practical value and may rather indicate sequencing and/or assembly aberrations. Nonetheless, users who is interested particularly in the fractured sequences may dig into the “domains only” output for the toxins that failed to pass the final filtering. The decent number of novel toxins revealed in the preliminary studies highlight the necessity of further screening studies not only to expand the scopes of *Bt* nomenclature, but also to enrich the arsenal of biopesticidal agents. 

We performed the verification of how accurately CryProcessor predicts the positions of the domains among 3d-Cry toxin sequences. To carry out this procedure, we summarized the coordinates of start and stop positions of the domains obtained from CryProcessor and CryGetter, respectively [11]. We then calculated the mean differences between corresponding positions. The mean deviation reached 2.8 b.p. (Table S4). Mainly, discrepancies between the start positions of domains I and III contribute to the total deviation (6.28 and 7.38 b.p., respectfully), while for the rest of the coordinates, mean deviation did not exceed 1 (0.79 b.p.). However, manual queries in the Pfam database confirmed CryProcessor-generated layout. 

## 3. Discussion

In the present work, we described a novel tool for searching for Cry toxin sequences. The main advantage of CryProcessor algorithms is the domain structure validation. As it was shown in comparison with BtToxin_scanner [10], applying mining without proper verification may provide false positive results. By way of illustration, the OTW84533.1 protein which lacked second and third domains, responsible for receptor recognition in the gut of insects, is marked as 99.53% identical to the Cry9Da1 toxin via BtToxin_scanner [10]. Nonetheless, this entry is a partial sequence, presumably a sequence artifact. On the contrary, CryProcessor is capable of filtering false positive results originating from sequencing and assembly errors.

Another important advantage of CryProcessor is the potentially unlimited query size. The online version of BtToxin_scanner [10] is constrained to 16 MB, and the local version of this tool lacks in speed, used database novelty, and sensitivity. Therefore, CryProcessor can be utilized in large-scale scanning for Cry toxins among whole databases. It should be noted that, to our knowledge, the web version of CryProcessor is the first online tool that allows direct processing of raw Illumina reads to mine Cry toxin sequences. Last but not least advantage is the domain mappings, presented in the CryProcessor output. Manually curated models allow users to obtain the positions of the domains in analyzed toxins. CryGetter [11] possesses this ability only in terms of gathering Cry toxin data from *Bt* nomenclature, but it does not provide the opportunity to extract domains from the arbitrary query. 

In order to overcome difficulties of the emerging resistance to widely applied biopesticides [1], two plausible approaches could be proposed. First, it is possible to expand the frontiers of known toxins by the massive screening of huge databases. The second approach implies creating chimeric constructions by shuffling domains from different toxins. 

CryProcessor is capable of dealing successfully with both approaches. At this time, *Bt* nomenclature possesses 818 Cry toxins [5]; however, the NCBI Assembly contains 594 *Bt* genomes, the SRA archive contains 618 *Bt*-related entities, the IPG database comprises 664,912 *Bt* sequences, and the NCBI protein database includes 3,622,535 sequences, related to *B. thuringiensis*. As can be inferred from the benchmarking, CryProcessor has enough robustness and throughput to be used for large-scale searches. Moreover, using the SPAdes assembler [12] coupled with PathRacer [13] as underlying tools in the CryProcessor’s pipeline allows the processing of raw Illumina sequencing data in a highly efficient manner. Extracting toxins directly from assembly graphs with PathRacer has evidently shown its transcendence over analogues (Table 2).

Computational experiments with domain shuffling are only possible if a large amount of sequences of specific domains are presented. For now, there is no instrument that can possibly carry out this procedure. Manual queries using the Pfam database lack the potency to handle thousands of sequences while CryGetter [11] contains layout information only for a predefined number of toxins. Therefore, CryProcessor gives users a unique opportunity to obtain domain layout from potentially unlimited datasets.

To conclude, CryProcessor is a ready-to-implement, convenient, rapid, and robust instrument that outperforms its analogues and provides identification of a unique set of novel toxins, which can be further tested as potential sources of novel insecticides. It possesses unique novel features such as direct analysis of sequencing data and domain layout prediction, which makes it of great potential in expanding the scopes of databases and being applied in phylogenetic studies. 

## 4. Materials and Methods

### 4.1. The Pipeline General Overview

**Figure 1:**
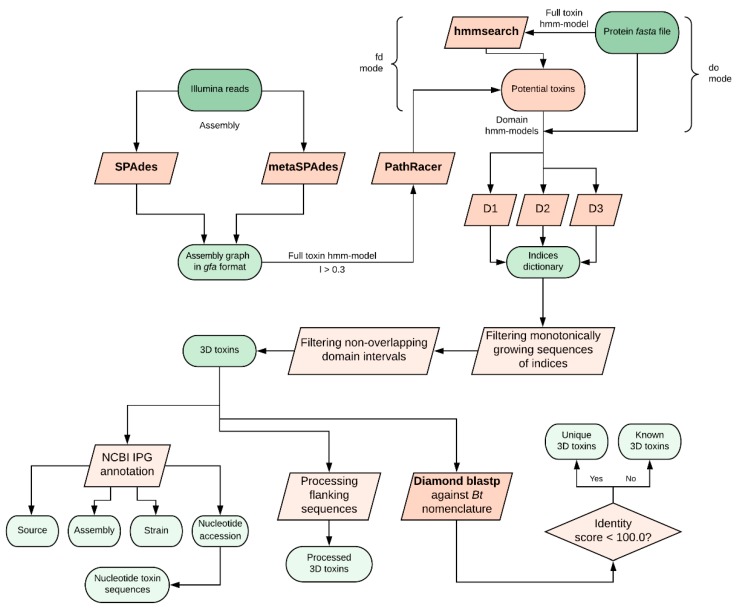
The detailed pipeline implemented in CryProcessor. All available modes of work are shown. Ovals denote data blocks, parallelograms denote processing steps, and rhombus indicates the decision-making step.

We developed a hidden Markov model (HMM)-based algorithm called CryProcessor for robust and effective Cry toxin mining from FASTA files or directly from Illumina FASTQ reads using PathRacer (v0.5, Center for Algorithmic Biotechnology, Saint-Petersburg, Russia, 2019) [13]. CryProcessor is a Python3.7-written tool with high-throughput capacity. The explicit manual is available at https://github.com/lab7arriam/cry_processor. The pipeline of the tool goes as follows (summarized in Figure 1):

1. Performing an HMM-based searching procedure against the query with hmmsearch (v3.3, Howard Hughes Medical Institute, Maryland, USA, 2019) [15] using either a full-toxin model (“find domains” mode) or three domain-specific models (“domains only” mode; see below);
2. Depending on the mode chosen in the previous step, either performing a domain mapping over found sequences and truncating accessory sequences (“find domains” mode) or filtering the sequences comprising all the three domains in the right sequential order (“domains only” mode);
3. Comparing found proteins to *Bt* nomenclature database [16] using Diamond (v0.9.25, Department of Computer Science and Center for Bioinformatics, University of Tübingen, Tübingen, Germany,2019) [17] with 100% sequence matching threshold;
4. Annotating the observed toxins against the Bt-related identical protein group (IPG) database entries.

### 4.2. HMM Model Composition

For HMM model compilation, sequences from CryGetter (v1.03, Instituto Federal de Educação, Ciência e Tecnologia de São Paulo, São Paulo, Brazil, 2016) [11] technical data were used. All four models (one for full-size toxins and one per each domain within mature Cry toxins) were made of the CryGetter-provided dummies with the hmmbuild utility and tested independently with 30% identity threshold to assess the accuracy of the search. 

### 4.3. Searching Modes

CryProcessor supports two search modes: the “domains only” mode and the “find domains” mode. The “find domains” mode implies the aforementioned screening strategy on four HMM-based models (three domain-restricted models and one full-size toxin model, respectively). The “domains only” mode implicates running hmmsearch with the domain models directly on the query without preceding filtration. Thus, in the case of the “domains only” mode, hmmsearch is performed on the full query within each searching iteration, while in the “find domains” mode, domain mining is executed on the sequences that passed the previous HMM-based filtration with full toxin models. When extracting 3d toxins, the sequence passes the filter only if the domain indices increase monotonously, indicating the right domain order, and the domain intervals do not overlap.

### 4.4. Toxin Annotation

The presence of Genbank accession numbers in the entry IDs allows CryProcessor to perform the annotation using efetch. The output represents a tab-separated (TSV) table with the accession numbers of the respective Strain, Assembly, and Nucleotide entries.

### 4.5. Assembly Mode

CryProcessor can be launched on Illumina reads in FASTQ format. SPAdes (v3.13.1, Center for Algorithmic Biotechnology, Saint-Petersburg, Russia, 2019) [12] or metaSPAdes (v3.13.1, Center for Algorithmic Biotechnology, Saint-Petersburg, Russia, 2019) [18] assemblers are implemented to get the assembly graph. Later, PathRacer [13] runs on the assembly graph in Graphical Fragment Assembly (GFA) format with a 30% similarity threshold. Afterwards, CryProcessor in “domains only” mode is launched against the mined sequences.

### 4.6. Web Implementation

For the sake of convenience, we also elaborated a web interface, comprising several key features of CryProcessor with some limitations. The web server includes the “domains only” mode. The IPG-annotation step in the online version is depreciated. Users can upload protein FASTA-files or Illumina reads in FASTQ-format. The uploading files are limited to 400 Mb. After the successful performance of tasks, the results of processing are sent to the user’s e-mail. 

### 4.7. Availability

CryProcessor 1.0 is a Linux command-line Python3.7 application. The software is licensed under the GNU General Public License v3.0, and its source code is free and available at https://github.com/lab7arriam/cry_processor. An automatically updating version of CryProcessor is also presented in Docker Hub, called lab7arriam/cry_processor. The web server is free for academic use and available upon registration at https://lab7.arriam.ru/tools/cry_processor.

## Figures and Tables

**Table 1:** Benchmarking of CryProcessor and BtToxin_scanner on the protein sequences of 511 *Bt* assemblies in FASTA format.

Tool	System Time	User Time	Real Time	Number of Toxins Found
CryProcessor (“domain only” mode)	67.20 s	42611.29 s	2902.15 s	602 (3d-Cry) 202 (new)
CryProcessor (“find domains” mode)	39.23 s	22186.64 s	1379.77 s	590 (3d-Cry) 199 (new)
BtToxin_scanner	241.79 s	27007.7 s	15822.07 s	419 (3d-Cry) 326 (new) *

The test dataset comprised all the *Bt* entries from the NCBI Assembly (511 genomes, 2810060 FASTA protein entries). * Only 128 of these proteins were marked as new after performing CryProcessor on the BtToxin_scanner output.

**Table 2:** Benchmarking of CryProcessor and BtToxin_scanner on the FASTA input.

Tool	System Time	User Time	Real Time	Number of Toxins Found
CryProcessor (“PathRacer” mode)	1636.47 s	31369.38 s	7723.37 s	5 (3d-Cry)*
BtToxin_scanner (“assembly” mode)	26301.08 s	23038.92 s	4561.89 s	no toxins found

For the test, a SRX2330733 submission from NCBI Sequence Read Archive (SRA) was used (BioProject: PRJNA352636; shotgun sequencing data for *Bt* strain HD-133; 3.2 Gb). * Detected 3d-Cry toxins: Cry9Ea1, Cry1Ia14, Cry1Ca5, Cry1Da1, Cry2Ab16.

**Table 3:** Negative control of CryProcessor and BtToxin_scanner.

Tool	System Time	User Time	Real Time	Number of Toxins Found
CryProcessor (“PathRacer” mode)	1490.93 s	6232.63 s	4491.51 s	no toxins found
BtToxin_scanner (“assembly” mode)	22333.75 s	18020.30 s	2075.42 s	no toxins found

## Supplementary Materials

The following are available online at https://www.mdpi.com/2072-6651/12/3/204/s1, Table S1: The full list of used assemblies for the benchmarking procedure, Table S2: Comparison of the “find domains” mode of CryProcessor to an online service of BtToxin_scanner [10] on the five assemblies of *Bt* genomes from the NCBI Assembly database, Table S3: Comparison of CryProcessor in the *fd* mode and BtToxin_scanner database and pipeline, Table S4: Comparison of start and stop positions of the domains obtained from CryGetter and CryProcessor.
