# Supplementary material for: No More Tears: Mining Sequencing Data for Novel Bt Cry Toxins with CryProcessor
Source: Toxins (Basel). 2020 Mar 23;12(3):204. doi: 10.3390/toxins12030204 (PMC7150774; doi:10.3390/toxins12030204)
Supplement: Supplementary file 1 [file toxins-12-00204-s001.zip › toxins-694813-SI.pdf]

Supplementary Materials: No More Tears: Mining Sequencing Data for Novel *Bt* Cry Toxins with CryProcessor

Anton E. Shikov, Yury V. Malovichko, Rostislav K. Skitchenko, Anton A. Nizhnikov and Kirill S. Antonets

Table 1. The full list of assemblies used for the benchmarking procedure.

GCA_000008505.1	GCA_002561795.1	GCA_002236945.1	GCA_002911795.1	GCA_002570625.1
GCA_000015065.1	GCA_002561825.1	GCA_002243685.1	GCA_002911825.1	GCA_002570635.1
GCA_000092165.1	GCA_002562035.1	GCA_002268645.2	GCA_002911835.1	GCA_002570705.1
GCA_000161475.1	GCA_002562045.1	GCA_002385135.1	GCA_002911865.1	GCA_002570735.1
GCA_000161495.1	GCA_002562055.1	GCA_002550055.1	GCA_002911875.1	GCA_002570815.1
GCA_000161515.1	GCA_002562205.1	GCA_002550185.1	GCA_002911905.1	GCA_002570855.1
GCA_000161535.1	GCA_002562295.1	GCA_002550215.1	GCA_002911925.1	GCA_002570885.1
GCA_000161555.1	GCA_002562415.1	GCA_002550295.1	GCA_002911945.1	GCA_002570925.1
GCA_000161575.1	GCA_002562425.1	GCA_002550455.1	GCA_002911955.1	GCA_002570955.1
GCA_000161595.1	GCA_002562665.1	GCA_002550605.1	GCA_002911965.1	GCA_002571025.1
GCA_000161615.1	GCA_002562915.1	GCA_002550745.1	GCA_002911975.1	GCA_002571105.1
GCA_000161635.1	GCA_002562975.1	GCA_002550785.1	GCA_002912025.1	GCA_002571115.1
GCA_000161655.1	GCA_002563045.1	GCA_002550915.1	GCA_002912045.1	GCA_002571245.1
GCA_000161675.1	GCA_002563145.1	GCA_002550955.1	GCA_002912065.1	GCA_002571265.1
GCA_000161695.1	GCA_002563165.1	GCA_002551615.1	GCA_002912075.1	GCA_002571305.1
GCA_000161715.1	GCA_002563205.1	GCA_002551695.1	GCA_002912105.1	GCA_002571315.1
GCA_000161735.1	GCA_002563275.1	GCA_002551805.1	GCA_002912115.1	GCA_002572135.1
GCA_000167695.1	GCA_002564405.1	GCA_002551885.1	GCA_002912125.1	GCA_002573505.1
GCA_000190515.1	GCA_002564505.1	GCA_002551895.1	GCA_002912135.1	GCA_002574095.1
GCA_000193355.1	GCA_002564545.1	GCA_002551955.1	GCA_002912215.1	GCA_002574115.1
GCA_000292455.1	GCA_002564645.1	GCA_002552035.1	GCA_002921215.1	GCA_002574125.1
GCA_000292705.1	GCA_002564955.1	GCA_002552095.1	GCA_002969075.1	GCA_002574265.1
GCA_000300475.1	GCA_002565085.1	GCA_002552245.1	GCA_002994815.1	GCA_002574295.1
GCA_000306745.1	GCA_002565105.1	GCA_002552325.1	GCA_002998815.1	GCA_002574325.1
GCA_000338755.1	GCA_002565195.1	GCA_002552395.1	GCA_002998855.1	GCA_002574365.1
GCA_000341665.1	GCA_002565265.1	GCA_002552415.1	GCA_003046465.1	GCA_002574435.1
GCA_000342025.1	GCA_002565385.1	GCA_002552905.1	GCA_003054785.1	GCA_002574475.1
GCA_000387405.2	GCA_002565435.1	GCA_002552915.1	GCA_003112375.1	GCA_002574495.1
GCA_000468975.2	GCA_002565845.1	GCA_002552985.1	GCA_003150715.1	GCA_002574505.1
GCA_000468995.3	GCA_002565905.1	GCA_002553035.1	GCA_003312445.1	GCA_002574575.1
GCA_000497525.2	GCA_002565945.1	GCA_002553045.1	GCA_003399465.1	GCA_002574595.1
GCA_000500585.1	GCA_002566135.1	GCA_002553055.1	GCA_003408695.1	GCA_002574645.1
GCA_000503755.1	GCA_002566145.1	GCA_002553105.1	GCA_003444735.1	GCA_002574675.1
GCA_000530375.1	GCA_002566295.1	GCA_002553205.1	GCA_003445395.1	GCA_002574695.1
GCA_000571955.1	GCA_002566325.1	GCA_002553315.1	GCA_003546665.1	GCA_002574725.1
GCA_000585975.1	GCA_002566355.1	GCA_002555365.1	GCA_003612265.1	GCA_002574735.1
GCA_000600315.1	GCA_002566395.1	GCA_002558015.1	GCA_003626955.1	GCA_002574805.1
GCA_000688795.1	GCA_002566485.1	GCA_002558035.1	GCA_003719805.1	GCA_002574895.1
GCA_000710255.1	GCA_002566545.1	GCA_002558075.1	GCA_003991175.1	GCA_002574935.1
GCA_000717535.1	GCA_002566555.1	GCA_002558235.1	GCA_004009875.1	GCA_002575015.1
GCA_000747545.1	GCA_002566645.1	GCA_002558335.1	GCA_004153515.1	GCA_002575035.1
GCA_000774075.2	GCA_002566715.1	GCA_002558355.1	GCA_004167115.1	GCA_002575075.1
GCA_000803665.1	GCA_002566935.1	GCA_002558385.1	GCA_004194435.2	GCA_002575195.1
GCA_000816555.1	GCA_002567205.1	GCA_002558525.1	GCA_004194455.1	GCA_002575305.1
GCA_000832485.1	GCA_002567265.1	GCA_002558545.1	GCA_004194475.1	GCA_002575325.1

GCA_000832825.1	GCA_002567295.1	GCA_002558555.1	GCA_004309715.1	GCA_002575365.1
GCA_000832925.1	GCA_002567585.1	GCA_002558595.1	GCA_004330315.1	GCA_002575395.1
GCA_000833085.1	GCA_002567865.1	GCA_002558635.1	GCA_004330415.1	GCA_002575445.1
GCA_000833655.1	GCA_002567875.1	GCA_002558795.1	GCA_004346705.1	GCA_002575615.1
GCA_000835025.1	GCA_002567905.1	GCA_002558815.1	GCA_004354265.1	GCA_002575645.1
GCA_000835235.1	GCA_002567945.1	GCA_002558915.1	GCA_004368515.1	GCA_002575705.1
GCA_000878525.1	GCA_002567955.1	GCA_002558995.1	GCA_004519705.1	GCA_002575895.1
GCA_000940785.1	GCA_002567995.1	GCA_002559295.1	GCA_900094685.1	GCA_002575925.1
GCA_000948155.1	GCA_002568045.1	GCA_002559535.1	GCA_900108905.1	GCA_002576025.1
GCA_000948235.1	GCA_002568055.1	GCA_002559825.1	GCA_002560645.1	GCA_002576055.1
GCA_000948325.1	GCA_002568085.1	GCA_002559855.1	GCA_002560695.1	GCA_002576125.1
GCA_000969665.1	GCA_002568175.1	GCA_002559875.1	GCA_002560805.1	GCA_002576135.1
GCA_001017635.1	GCA_002568285.1	GCA_002559955.1	GCA_002560815.1	GCA_002576375.1
GCA_001182785.1	GCA_002568345.1	GCA_002560005.1	GCA_002560855.1	GCA_002576405.1
GCA_001183785.1	GCA_002568385.1	GCA_002560055.1	GCA_002560875.1	GCA_002576485.1
GCA_001238465.1	GCA_002568515.1	GCA_002560305.1	GCA_002560885.1	GCA_002576565.1
GCA_001276195.1	GCA_002568525.1	GCA_002560335.1	GCA_002560925.1	GCA_002576585.1
GCA_001296435.1	GCA_002568585.1	GCA_002560455.1	GCA_002561045.1	GCA_002576645.1
GCA_001402735.1	GCA_002568595.1	GCA_002560465.1	GCA_002561175.1	GCA_002576655.1
GCA_001420855.1	GCA_002569275.1	GCA_002560515.1	GCA_002561275.1	GCA_002576665.1
GCA_001455345.1	GCA_002569645.1	GCA_002560605.1	GCA_002561335.1	GCA_002576755.1
GCA_001517195.1	GCA_002569825.1	GCA_002561585.1	GCA_002561375.1	GCA_002576765.1
GCA_001548175.1	GCA_002570475.1	GCA_002561645.1	GCA_002561465.1	GCA_002576875.1
GCA_001573165.1	GCA_002570505.1	GCA_002561665.1	GCA_002561475.1	GCA_002576925.1
GCA_001595725.1	GCA_002570585.1	GCA_002561735.1	GCA_002585935.1	GCA_002577005.1
GCA_001598095.1	GCA_002147575.1	GCA_002146695.1	GCA_002586085.1	GCA_002577025.1
GCA_001618665.1	GCA_002147605.1	GCA_002146705.1	GCA_002589015.1	GCA_002577255.1
GCA_001640965.1	GCA_002147635.1	GCA_002146725.1	GCA_002589045.1	GCA_002577325.1
GCA_001675515.1	GCA_002147645.1	GCA_002146755.1	GCA_002795225.1	GCA_002577445.1
GCA_001677055.1	GCA_002147655.1	GCA_002146785.1	GCA_002893505.1	GCA_002577525.1
GCA_001685565.1	GCA_002147685.1	GCA_002146815.1	GCA_002893515.1	GCA_002577565.1
GCA_001692675.1	GCA_002147715.1	GCA_002146825.1	GCA_002893525.1	GCA_002577605.1
GCA_001721165.1	GCA_002147725.1	GCA_002146845.1	GCA_002893565.1	GCA_002577625.1
GCA_001757675.1	GCA_002147745.1	GCA_002146865.1	GCA_002893585.1	GCA_002577805.1
GCA_001757685.1	GCA_002147755.1	GCA_002146885.1	GCA_002893605.1	GCA_002577835.1
GCA_001757695.1	GCA_002147795.1	GCA_002146905.1	GCA_002911745.1	GCA_002578165.1
GCA_001757745.1	GCA_002147805.1	GCA_002146925.1	GCA_002911755.1	GCA_002578625.1
GCA_001757755.1	GCA_002147825.1	GCA_002146945.1	GCA_002911785.1	GCA_002581075.1
GCA_001757775.1	GCA_002147835.1	GCA_002146975.1	GCA_002147395.1	GCA_002582575.1
GCA_001880115.1	GCA_002147895.1	GCA_002146985.1	GCA_002147415.1	GCA_002582685.1
GCA_001880125.1	GCA_002147905.1	GCA_002147005.1	GCA_002147425.1	GCA_002582925.1
GCA_001883875.1	GCA_002147935.1	GCA_002147025.1	GCA_002147435.1	GCA_002583105.1
GCA_001883945.1	GCA_002147955.1	GCA_002147055.1	GCA_002147475.1	GCA_002583235.1
GCA_001968805.1	GCA_002147965.1	GCA_002147085.1	GCA_002147495.1	GCA_002583285.1
GCA_002024545.1	GCA_002147985.1	GCA_002147105.1	GCA_002147505.1	GCA_002583445.1
GCA_002025105.1	GCA_002148015.1	GCA_002147125.1	GCA_002147535.1	GCA_002583505.1
GCA_002119445.1	GCA_002148025.1	GCA_002147155.1	GCA_002147555.1	GCA_002583895.1
GCA_002146315.1	GCA_002148045.1	GCA_002147165.1	GCA_002147565.1	GCA_002584055.1
GCA_002146325.1	GCA_002148065.1	GCA_002147185.1	GCA_002146495.1	GCA_002584185.1
GCA_002146335.1	GCA_002148095.1	GCA_002147235.1	GCA_002146505.1	GCA_002584205.1
GCA_002146345.1	GCA_002148105.1	GCA_002147245.1	GCA_002146525.1	GCA_002584325.1
GCA_002146365.1	GCA_002148135.1	GCA_002147255.1	GCA_002146545.1	GCA_002584605.1
GCA_002146395.1	GCA_002148155.1	GCA_002147295.1	GCA_002146595.1	GCA_002584855.1
GCA_002146405.1	GCA_002173755.1	GCA_002147315.1	GCA_002146605.1	GCA_002584925.1
GCA_002146425.1	GCA_002184245.1	GCA_002147325.1	GCA_002146615.1	GCA_002585315.1
GCA_002146445.1	GCA_002192515.1	GCA_002147335.1	GCA_002146625.1	GCA_002585635.1

GCA_002146465.1	GCA_002222555.1	GCA_002147375.1	GCA_002146655.1	GCA_002585795.1
GCA_002146665.1				

Table S2. Comparison of find domains mode of CryProcessor with an online service of BtToxin_scanner on the five assemblies of *Bt* genomes from NCBI Assembly database. Domain structure screens were taken from Pfam database.

1) Assembly: GCA_002146445.

Accession	CryProcessor		BtToxin_Scanner	
	Top hit	Identity	Top hit	Identity
OTW84525.1	Cry1Ie2	100	Cry1Ie1	95.97
OTW84402.1	Cry1Na1	100	Cry1Ca5	51.54
OTW84346.1	Cry9Da4	99.7	Cry9Da1	99.53
OTW81421.1	Cry9Eb3	95.6	Cry9Eb1	94.97
OTW84522.1	–	–	Cry9Da1	99.53
OTW84533.1	–	–	Cry9Da1	99.53

Model for OTW84522.1, OTW84533.1:

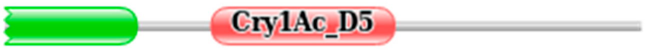

Model OTW84525.1, OTW84346.1, OTW84346.1 and OTW81421.1:

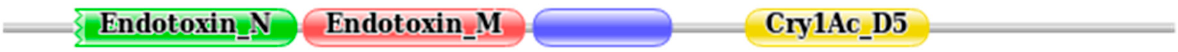

2) Assembly: GCA_000161475

Accession	CryProcessor		BtToxin_Scanner	
	Top hit	Identity	Top hit	Identity
EEM19417.1	Cry67Aa2	100	Cry67Aa2	100
EEM19405.1	Cry7Ba1	85.6	Cry7Ba1	85.6
EEM19403.1	Cry7Ea2	100	Cry7Ea3	100
EEM19308.1	Cry61Aa3	100	Cry61Aa3	100
EEM19090.1	Cry7Fa2	99.9	Cry7Fa2	99.91
EEM19459.1	–	–	Cry7Ca1	94.31

EEM19459.1: no domains found.

3) Assembly: GCA_000161715.

Accession	CryProcessor		BtToxin_Scanner	
	Top hit	Identity	Top hit	Identity
EEM92924.1	Cry1Hb1	100	Cry1Hb1	100
EEM92927.1	Cry1Bb1	99.9	Cry1Bb1	99.92
EEM92934.1	Cry1Ab18	100	Cry1Ab18	100
EEM92941.1	Cry1Nb1	100	Cry1Ca5	54.08
EEM92947.1	Cry1Ja2	100	Cry1Ja1	99.57
EEM92952.1	Cry1Id1	100	Cry1Id1	100
EEM92953.1	Cry1Da3	99.9	Cry1Da2	96.82
EEM92570.1	Cry8Aa1	47.1	Cry8Ba1	46.58
EEM93055.1	Cry2Ad1	100	Cry2Ad1	100
EEM92620.1	–	–	Cry6Aa3	28.45
EEM93105.1	–	–	Cry11Ba1	24.62
EEM92191.1	–	–	NO	NO

EM92620.1 model:

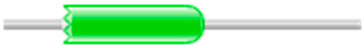

EEM93105.1 model:

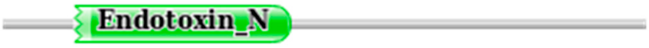

EEM92191.1: no domains found.

4) Assembly: GCA_002146755

Accession	CryProcessor		BtToxin_Scanner	
	Top hit	Identity	Top hit	Identity
OTY10661.1	Cry7Ka1	100	Cry7Ab4	67.84
OTY00887.1	Cry7Cb1	60.4	Cry7Ba1	57.07
OTY00858.1	Cry7Gc1	99.8	Cry7Ba1	59.77
OTY09875.1	–	–	Cry7Ba1	95.72
OTY10663.1	–	–	Cry7Ba1	95.72

OTY09875.1 and OTY10663.1: no domains found.

5) Assembly: GCA_002893565.

Accession	CryProcessor		BtToxin_Scanner	
	Top hit	Identity	Top hit	Identity
PNK41404.1	Cry1Ka2	100	Cry1Ka1	99.16
PNK40066.1	Cry1Fb3	99.9	Cry1Fb4	99.78
PNK40029.1	Cry1Da3	99.9	Cry1Da2	98.69
PNK40024.1	Cry1Ib11	99.9	Cry1Ib9	98.75
PNK40012.1	Cry1Ab18	100	Cry1Ab18	100
PNK46490.1	Cry1Bb1	99.9	Cry1Bb1	99.91
PNK46492.1	Cry1Hb1	100	Cry1Hb1	100
PNK42250.1	Cry1Id1	100	Cry1Id1	100
PNK42253.1	Cry1Ja2	100	Cry1Ja1	99.57
PNK42258.1	Cry1Nb1	100	Cry1Ca5	54.08
PNK36334.1	–	–	Cry2Ad1	100
PNK36450.1	–	–	Cry1Db1	100
PNK37031.1	–	–	Cry1Ga1	90.18
PNK38095.1	–	–	Cry2Aa9	100
PNK40094.1	–	–	Cry1Ab18	100
PNK41461.1	–	–	Cry2Ad1	100
PNK50401.1	–	–	NO	NO

PNK36334.1 model:

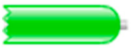

PNK38095.1 model:

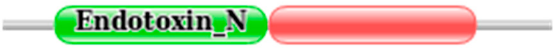

PNK41461.1 model:

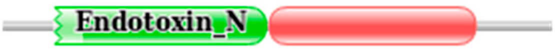

PNK36450.1, PNK37031.1, PNK40094.1 and PNK50401.1: no domains found.

Table S3. Comparison of CryProcessor in fd mode with BtToxin_scanner database and pipeline. The comparison was performed on all *Bt* genomes from NCBI Assembly database. The last two columns denote CryProcessor output with the database taken from the standalone version of BtToxin_scanner.

Accession	CryProcessor (native db)		BtToxin_scanner (standalone)		BtToxin_scanner (online)		CryProcessor (BtToxin_scanner db)	
	Top Hit	Identity	Top Hit	Identity	Top Hit	Identity	Top Hit	Identity
EEM19417.1	Cry67Aa2	100.0	Cry67Aa2	100.00	Cry67Aa2	100.00	Cry67Aa2	100.0
EEM19403.1	Cry7Ea2	100.0	Cry7Ea3	100.00	Cry7Ea3	100.00	Cry7Ea3	100.0
EEM19308.1	Cry61Aa3	100.0	Cry61Aa3	100.00	Cry61Aa3	100.00	Cry61Aa3	100.0
EEM19090.1	Cry7Fa2	99.9	Cry7Fa2	99.91	Cry7Fa2	100.00	Cry7Fa2	99.9
EEM32076.1	Cry1Ab12	100.0	Cry1Ab9	100.00	Cry1Ab5	100.00	Cry1Ab1	100.0
EEM31561.1	Cry1Ba1	100.0	Cry1Ba5	100.00	Cry1Ba5	100.00	Cry1Ba1	100.0
EEM38018.1	Cry1Aa13	100.0	Cry1Aa9	100.00	Cry1Aa9	100.00	Cry1Aa13	100.0
EEM44869.1	Cry5Ca1	100.0	Cry5Ca2	100.00	*	*	Cry5Ca2	100.0
EEM44456.1	Cry65Aa2	99.9	Cry65Aa2	99.91	Cry65Aa2	100.00	Cry65Aa2	99.9
EEM44315.1	Cry5Da1	100.0	Cry5Da2	100.00	*	*	Cry5Da2	100.0
EEM44256.1	Cry5Ea2	100.0	Cry5Ea2	100.00	*	*	Cry5Ea2	100.0
EEM50110.1	Cry1Ac10	100.0	Cry1Ac9	100.00	Cry1Ac9	100.00	Cry1Ac1	100.0
EEM55953.1	Cry7Ab4	99.8	Cry7Ab4	99.82	Cry7Ab4	100.00	Cry7Ab4	99.8
EEM62552.1	Cry1Ba1	100.0	Cry1Ba5	100.00	Cry1Ba5	100.00	Cry1Ba1	100.0
EEM62204.1	Cry1Ab12	100.0	Cry1Ab9	100.00	Cry1Ab5	100.00	Cry1Ab1	100.0
EEM68667.1	Cry41Ba2	100.0	Cry41Ba2	100.00	Cry41Ba2	100.00	Cry41Ba2	100.0
EEM68355.1	Cry41Ba1	100.0	Cry66Aa2	100.00	Cry66Aa2	100.00	Cry66Aa2	100.0
EEM86551.1	Cry8Ma3	100.0	Cry8−like	100.00	Cry8Ma2	100.00	Cry8−like	100.0
EEM93055.1	Cry2Ad1	100.0	Cry2Ad1	100.00	*	*	Cry2Ad1	100.0
EEM92924.1	Cry1Hb1	100.0	Cry1Hb1	100.00	Cry1Hb1	100.00	Cry1Hb1	100.0
EEM92927.1	Cry1Bb1	99.9	Cry1Bb1	99.92	Cry1Bc1	100.00	Cry1Bb1	99.9
EEM92934.1	Cry1Ab18	100.0	Cry1Ab18	100.00	Cry1Ab18	100.00	Cry1Ab18	100.0
EEM92941.1	Cry1Nb1	100.0	−	−	Cry1Ca4	52.73	PesticidalProtein	100.0
EEM92947.1	Cry1Ja2	100.0	Cry1Ja1	99.57	Cry1Ja1	99.09	Cry1Ja1	99.6
EEM92952.1	Cry1Id1	100.0	Cry1Id1	100.00	Cry1Id1	100.00	Cry1Id1	100.0
EEM92953.1	Cry1Da3	99.9	Cry1Da2	96.82	Cry1Da2	99.82	Cry1Da1	96.8
EEM92570.1	Cry8Aa1	47.1	Cry8Ba1	46.58	Cry8Aa1	47.00	Cry8Aa1	47.1
EEM99282.1	Cry10Aa3	100.0	Cry10Aa4	100.00	Cry10Aa4	100.00	Cry10Aa3	100.0
EEM99034.1	Cry11Aa4	100.0	Cry11Aa4	100.00	*	*	Cry11Aa4	100.0
ADY24756.1	Cry26Aa1	95.4	−	−	Cry26Aa1	100.00	Cry26Aa1	95.4
ADY24870.1	Cry28Aa2	99.7	Cry28Aa2	99.73	Cry28Aa2	99.47	Cry28Aa2	99.7
ADY25012.1	Cry28Aa2	99.6	Cry28Aa2	99.65	Cry28Aa2	99.47	Cry28Aa2	99.6
AEA19203.1	Cry1Ba1	99.9	Cry1Ba5	99.92	Cry1Ba5	100.00	Cry1Ba1	99.9
AEA19603.1	Cry2Ab16	100.0	Cry2Ab3	100.00	*	*	Cry2Ab1	100.0
AEA19608.1	Cry1Aa12	100.0	Cry1Aa3	100.00	Cry1Aa3	100.00	Cry1Aa12	100.0
AEA19609.1	Cry1Ia14	100.0	Cry1Ia2	100.00	Cry1Ia2	100.00	Cry1Ia10	100.0
AEA19615.1	Cry2Aa1	100.0	Cry2Aa9	100.00	*	*	Cry2Aa1	100.0
AFQ19845.1	Cry1Aa13	100.0	Cry1Aa9	100.00	Cry1Aa9	100.00	Cry1Aa13	100.0
AFQ30496.1	Cry4Ba1	100.0	Cry4Ba5	100.00	cry4Ba	100.00	Cry4Ba	100.0
AFQ30505.1	Cry11Aa1	100.0	Cry11Aa3	100.00	*	*	Cry11Aa1	100.0
AFQ30558.1	Cry4Aa2	100.0	Cry4Aa4	100.00	Cry4Aa4	100.00	Cry4Aa2	100.0
AFQ30560.1	Cry10Aa3	100.0	Cry10Aa4	100.00	Cry10Aa4	100.00	Cry10Aa3	100.0
AFU17214.1	Cry69Aa2	100.0	Cry43Ba1	38.18	Cry7Da1	32.45	Cry4Ba	37.8
AFU17297.1	Cry54Aa1	100.0	Cry54Aa1	100.00	Cry54Aa1	100.00	Cry54Aa1	100.0
AFU17323.1	Cry68Aa1	99.9	Cry24Ba1	31.68	Cry9Aa2	36.17	Cry9Aa1	32.6
AFU17333.1	Cry30Fa1	98.8	Cry30Fa1	98.84	Cry30Fa1	99.15	Cry30Fa1	98.8
AFU17344.1	Cry54Aa1	88.1	Cry54Aa1	84.47	Cry54Aa1	84.00	Cry54Aa1	88.1
AFU17354.1	Cry53Ab1	99.7	Cry53Ab1	99.70	Cry53Ab1	100.00	Cry53Ab1	99.7
AFU16949.1	Cry69Aa1	100.0	Cry4Ba5	38.98	Cry7Da1	32.45	Cry4Ba	39.2
AFU17015.1	Cry4Cc1	99.4	Cry4Cc1	99.39	Cry4Cc1	98.92	Cry4Cc1	99.4
AFU17024.1	Cry70Ba1	98.6	Cry31Aa6−like	66.10	*	*	Cry31Aa6−like	64.9

AGE81486.1	Cry1Ac10	100.0	Cry1Ac9	100.00	Cry1Ac9	100.00	Cry1Ac1	100.0
AGG04729.1	Cry1Ba1	99.9	Cry1Ba5	99.92	Cry1Ba5	100.00	Cry1Ba1	99.9
AGG05318.1	Cry2Ab16	100.0	Cry2Ab3	100.00	*	*	Cry2Ab1	100.0
AGG05325.1	Cry1Aa12	100.0	Cry1Aa3	100.00	Cry1Aa3	100.00	Cry1Aa12	100.0
AGG05326.1	Cry1Ia14	100.0	Cry1Ia2	100.00	Cry1Ia2	100.00	Cry1Ia10	100.0
AGG05333.1	Cry2Aa1	100.0	Cry2Aa9	100.00	*	*	Cry2Aa1	100.0
AGG04467.1	Cry1Ab21	100.0	Cry1Ab21	100.00	Cry1Ab21	100.00	Cry1Ab21	100.0
ERH96459.1	Cry1Aa12	100.0	Cry1Aa3	100.00	Cry1Aa3	100.00	Cry1Aa12	100.0
ERH97545.1	Cry2Ab16	100.0	Cry2Ab3	100.00	*	*	Cry2Ab1	100.0
ERH97550.1	Cry1Ba1	99.8	Cry1Ba5	99.84	Cry1Ba5	100.00	Cry1Ba1	99.8
ERH97551.1	Cry1Ia14	100.0	Cry1Ia2	100.00	Cry1Ia2	100.00	Cry1Ia10	100.0
ERH97558.1	Cry2Aa1	100.0	Cry2Aa9	100.00	*	*	Cry2Aa1	100.0
ETE89313.1	Cry2Ab16	100.0	Cry2Ab3	100.00	*	*	Cry2Ab1	100.0
ETE91940.1	Cry1Da1	100.0	Cry1Da2	100.00	Cry1Da2	100.00	Cry1Da1	100.0
ETE91952.1	Cry1Ia14	100.0	Cry1Ia2	100.00	Cry1Ia2	100.00	Cry1Ia10	100.0
ETE96065.1	Cry9Ea1	100.0	Cry9Ea2	100.00	Cry9Ea2	100.00	Cry9Ea1	100.0
ETE88270.1	Cry1Fa1	100.0	Cry1Fa2	100.00	Cry1Fa2	100.00	Cry1Fa1	100.0
ETE89765.1	Cry1Ia7	100.0	Cry1Ia7	100.00	Cry1Ia7	100.00	Cry1Ia7	100.0
ETE89897.1	Cry1Da1	100.0	Cry1Da2	100.00	Cry1Da2	100.00	Cry1Da1	100.0
ETE93019.1	Cry9Ea1	100.0	Cry9Ea2	100.00	Cry9Ea2	100.00	Cry9Ea1	100.0
ETE96169.1	Cry1Ca8	97.8	Cry1Ca9	97.82	Cry1Ca9	100.00	Cry1Ca7	97.8
ETF00019.1	Cry1Aa12	95.9	–	–	*	*	Cry1Aa12	95.9
AHA70031.1	Cry5Ba2	100.0	Cry5Ba2	100.00	*	*	Cry5Ba2	100.0
CDN39519.1	Cry21Fa1	100.0	Cry21Ba1	53.58	*	*	Cry21Aa2	53.6
CDN39777.1	Cry21Ha1	100.0	Cry21Ba1	45.87	*	*	Cry21Ba1	45.9
EXY04803.1	Cry1Bd1	99.9	Cry1Bd1	99.92	Cry1Bd1	100.00	Cry1Bd1	99.9
EXY04555.1	Cry1Ab35	100.0	Cry1Ab18	97.70	Cry1Ab5	100.00	Cry1Ab18	97.4
EXY04526.1	Cry1Cb1	100.0	Cry1Cb2	100.00	Cry1Cb2	100.00	Cry1Cb2	100.0
EXY04520.1	Cry1Ga1	100.0	Cry1Ga1	100.00	Cry1Ga1	100.00	Cry1Ga1	100.0
EXY04476.1	Cry9Aa1	100.0	Cry9Aa1	100.00	Cry9Aa1	100.00	Cry9Aa1	100.0
EXY04468.1	Cry1Gb2	99.9	Cry1Gb2	99.91	Cry1Gb2	100.00	Cry1Gb2	99.9
EXY05450.1	Cry1Ib7	99.9	Cry1Ib3	99.86	Cry1Ib3	100.00	Cry1Ib3	99.9
EXY05451.1	Cry1Db1	100.0	Cry1Db1	100.00	Cry1Db2	100.00	Cry1Db1	100.0
EXY05452.1	Cry1Fb1	100.0	Cry1Fb5	100.00	Cry1Fb2	100.00	Cry1Fb1	100.0
EXY05361.1	Cry2Ac6	100.0	Cry2Ac6	100.00	*	*	Cry2Ac6	100.0
EXY05362.1	Cry2Ad2	98.1	Cry2Ad1	99.82	*	*	Cry2Ad2	98.1
EXY05115.1	Cry2Ab4	100.0	Cry2Ab4	100.00	*	*	Cry2Ab4	100.0
AHZ54986.1	Cry2Ab16	100.0	Cry2Ab3	100.00	*	*	Cry2Ab1	100.0
AHZ54991.1	Cry1Aa12	100.0	Cry1Aa3	100.00	Cry1Aa3	100.00	Cry1Aa12	100.0
AHZ54992.1	Cry1Ia14	100.0	Cry1Ia2	100.00	Cry1Ia2	100.00	Cry1Ia10	100.0
AHZ54998.1	Cry2Aa1	100.0	Cry2Aa9	100.00	*	*	Cry2Aa1	100.0
AHZ55258.1	Cry1Ac4	100.0	Cry1Ac4	100.00	Cry1Ac4	100.00	Cry1Ac4	100.0
KEH48722.1	Cry1Ac20	100.0	Cry1Ac5	100.00	Cry1Ac32	100.00	Cry1Ac17	100.0
KEH48187.1	Cry1Ia14	100.0	Cry1Ia2	100.00	Cry1Ia2	100.00	Cry1Ia10	100.0
KEH48195.1	Cry2Aa1	100.0	Cry2Aa9	100.00	*	*	Cry2Aa1	100.0
KEH48048.1	Cry2Ab16	100.0	Cry2Ab3	100.00	*	*	Cry2Ab1	100.0
KEH45261.1	Cry1Ab12	100.0	Cry1Ab9	100.00	Cry1Ab5	100.00	Cry1Ab1	100.0
AIE37434.1	Cry2Ab16	99.8	Cry2Ab3	99.84	*	*	Cry2Ab1	99.8
AIE37439.1	Cry1Aa11	99.9	Cry1Aa8	99.91	Cry1Aa5	100.00	Cry1Aa11	99.9
AIE37440.1	Cry1Ia14	100.0	Cry1Ia2	100.00	Cry1Ia2	100.00	Cry1Ia10	100.0
AIE37446.1	Cry2Aa1	99.4	Cry2Aa9	99.37	*	*	Cry2Aa1	99.4
AIE37011.1	Cry1Ab12	100.0	Cry1Ab9	100.00	Cry1Ab5	100.00	Cry1Ab1	100.0
AIE37211.1	Cry1Ac5	100.0	Cry1Ac5	100.00	Cry1Ac32	100.00	Cry1Ac17	100.0
AIM34735.1	Cry2Ab16	100.0	Cry2Ab3	100.00	*	*	Cry2Ab1	100.0
AIM34741.1	Cry1Aa12	100.0	Cry1Aa3	100.00	Cry1Aa3	100.00	Cry1Aa12	100.0
AIM34742.1	Cry1Ia14	100.0	Cry1Ia2	100.00	Cry1Ia2	100.00	Cry1Ia10	100.0
AIM34751.1	Cry2Aa1	100.0	Cry2Aa9	100.00	*	*	Cry2Aa1	100.0
AIM34400.1	Cry1Ac4	100.0	Cry1Ac4	100.00	Cry1Ac4	100.00	Cry1Ac4	100.0

AIM34414.1	Cry1Ac4	100.0	Cry1Ac4	100.00	Cry1Ac4	100.00	Cry1Ac4	100.0
ALL21863.1	Cry7Gd1	100.0	Cry7Ba1	60.94	Cry61Aa3	42.36	Cry7Ba1	61.0
ALL25005.1	Cry7Cb1	100.0	Cry7Ca1	75.58	Cry7Ca1	63.82	Cry7Ca1	74.4
ALL21966.1	Cry7Ea2	100.0	Cry7Ea3	100.00	Cry7Ea3	100.00	Cry7Ea3	100.0
ALL21973.1	Cry67Aa2	100.0	Cry67Aa2	100.00	Cry67Aa2	100.00	Cry67Aa2	100.0
ALL21979.1	Cry7Fa2	99.9	Cry7Fa2	99.91	Cry7Fa2	100.00	Cry7Fa2	99.9
ALL21984.1	Cry61Aa3	100.0	Cry61Aa3	100.00	Cry61Aa3	100.00	Cry61Aa3	100.0
ALL11724.1	Cry7Gd1	100.0	–	–	Cry61Aa3	42.36	Cry7Ba1	61.0
ALL11871.1	Cry7Cb1	100.0	–	–	Cry7Ca1	63.82	Cry7Ca1	74.4
ALL11828.1	Cry7Ea2	100.0	Cry7Ea3	100.00	Cry7Ea3	100.00	Cry7Ea3	100.0
ALL11835.1	Cry67Aa2	100.0	Cry67Aa2	100.00	Cry67Aa2	100.00	Cry67Aa2	100.0
ALL11841.1	Cry7Fa2	99.9	Cry7Fa2	99.91	Cry7Fa2	100.00	Cry7Fa2	99.9
ALL11846.1	Cry61Aa3	100.0	Cry61Aa3	100.00	Cry61Aa3	100.00	Cry61Aa3	100.0
AJA23643.1	Cry1Ac10	100.0	Cry1Ac9	100.00	Cry1Ac9	100.00	Cry1Ac1	100.0
AJA23501.1	Cry2Ab16	100.0	Cry2Ab3	100.00	*	*	Cry2Ab1	100.0
AJA23506.1	Cry1Aa12	100.0	Cry1Aa3	100.00	Cry1Aa3	100.00	Cry1Aa12	100.0
AJA23507.1	Cry1Ia14	100.0	Cry1Ia2	100.00	Cry1Ia2	100.00	Cry1Ia10	100.0
AJA23511.1	Cry9Ea1	100.0	Cry9Ea2	100.00	Cry9Ea2	100.00	Cry9Ea1	100.0
AJA23086.1	Cry1Ca8	100.0	Cry1Ca9	100.00	Cry1Ca9	100.00	Cry1Ca7	100.0
AJA23088.1	Cry1Da1	100.0	Cry1Da2	100.00	Cry1Da2	100.00	Cry1Da1	100.0
KIP24267.1	Cry1Ka1	98.4	Cry1Ka1	99.09	Cry1Ka1	98.94	Cry1Ka1	98.4
KIP24195.1	Cry1Ib11	99.9	Cry1Ib9	98.75	Cry1Ib6	100.00	Cry1Ib10	98.7
AJK38183.1	Cry1Ac5	100.0	Cry1Ac5	100.00	Cry1Ac32	100.00	Cry1Ac17	100.0
AJK37985.1	Cry2Aa1	100.0	Cry2Aa9	100.00	*	*	Cry2Aa1	100.0
AJK38154.1	Cry1Ia14	100.0	Cry1Ia2	100.00	Cry1Ia2	100.00	Cry1Ia10	100.0
AJK37978.1	Cry1Aa11	100.0	Cry1Aa8	100.00	Cry1Aa5	100.00	Cry1Aa11	100.0
AJK38083.1	Cry2Ab16	100.0	Cry2Ab3	100.00	*	*	Cry2Ab1	100.0
AJK38600.1	Cry1Ab12	100.0	Cry1Ab9	100.00	Cry1Ab5	100.00	Cry1Ab1	100.0
KIU76094.1	Cry65Aa2	99.9	Cry65Aa2	99.91	Cry65Aa2	100.00	Cry65Aa2	99.9
KIU74504.1	Cry5Ea2	100.0	Cry5Ea2	100.00	*	*	Cry5Ea2	100.0
KIU74325.1	Cry5Da1	100.0	Cry5Da2	100.00	*	*	Cry5Da2	100.0
KIU70667.1	Cry5Ca1	100.0	Cry5Ca2	100.00	*	*	Cry5Ca2	100.0
KKB28324.1	Cry2Ab16	100.0	Cry2Ab3	100.00	*	*	Cry2Ab1	100.0
KKB28329.1	Cry1Ac10	100.0	Cry1Ac9	100.00	Cry1Ac9	100.00	Cry1Ac1	100.0
AKJ62760.1	Cry1Ac5	100.0	Cry1Ac5	100.00	Cry1Ac32	100.00	Cry1Ac17	100.0
AKJ62786.1	Cry2Aa1	100.0	Cry2Aa9	100.00	*	*	Cry2Aa1	100.0
AKJ62789.1	Cry1Ia14	100.0	Cry1Ia2	100.00	Cry1Ia2	100.00	Cry1Ia10	100.0
AKJ62790.1	Cry1Aa11	100.0	Cry1Aa8	100.00	Cry1Aa5	100.00	Cry1Aa11	100.0
AKJ62795.1	Cry2Ab16	100.0	Cry2Ab3	100.00	*	*	Cry2Ab1	100.0
AKR12899.1	Cry30Ga2	97.1	Cry30Ga2	99.51	Cry30Ga2	99.47	Cry30Ga2	97.1
AKR12909.1	Cry71Aa1	100.0	Cry53Ab1	41.52	Cry53Ab1	40.73	Cry53Ab1	41.4
AKR12911.1	Cry72Aa1	100.0	Cry40Ba1	36.09	Cry40Ba1	36.78	Cry40Ba1	36.1
AKR12921.1	Cry70Aa1	99.9	Cry31Aa6−like	57.65	*	*	Cry31Aa6−like	57.6
AKR13091.1	Cry30Ea4	100.0	Cry30Ea2	99.56	Cry30Ea2	99.66	Cry30Ea2	99.6
AKR13097.1	Cry69Ab1	100.0	Cry4Cc1	39.35	Cry7Da1	32.16	Cry4Ba	39.0
AKR13106.1	Cry50Ba2	99.9	Cry50Ba2	99.85	Cry50Ba1	99.65	Cry50Ba2	99.9
AKR13252.1	Cry56Aa4	100.0	Cry56Aa2	99.85	Cry56Aa2	100.00	Cry56Aa2	99.8
AKR13360.1	Cry54Ba2	100.0	Cry54Ba1	99.57	Cry54Ba1	99.49	Cry54Ba1	99.6
AKR38906.1	Cry7Da1	100.0	Cry7Da1	100.00	Cry7Da1	100.00	Cry7Da1	100.0
AKR38911.1	Cry7Fb2	100.0	Cry7Fa2	76.93	Cry7Fa2	69.24	Cry7Fa2	77.0
AKR38949.1	Cry7Ga2	100.0	Cry7Da1	63.12	Cry61Aa3	42.36	Cry7Da1	63.3
KQB18012.1	Cry4Ba1	100.0	–	–	cry4Ba	100.00	Cry4Ba	100.0
KQB17992.1	Cry10Aa3	100.0	Cry10Aa4	100.00	Cry10Aa4	100.00	Cry10Aa3	100.0
KQB18067.1	Cry11Aa1	100.0	Cry11Aa3	100.00	*	*	Cry11Aa1	100.0
ALL62164.1	Cry1Ac5	100.0	Cry1Ac5	100.00	Cry1Ac32	100.00	Cry1Ac17	100.0
ALL62189.1	Cry2Aa1	100.0	Cry2Aa9	100.00	*	*	Cry2Aa1	100.0
BAR87544.1	Cry2Aa8	100.0	Cry2Aa8	100.00	*	*	Cry2Aa8	100.0
BAR87552.1	Cry1Ia12	100.0	Cry1Ia2	100.00	Cry1Ia5	100.00	Cry1Ia10	100.0

BAR87554.1	Cry1Ea11	98.2	Cry1Ea7	98.21	Cry1Ea5	100.00	Cry1Ea11	98.2
AMR06122.1	Cry8Ea3	100.0	Cry8Ea1	100.00	Cry8Ea1	100.00	Cry8Ea1	100.0
AMR06151.1	Cry8Ab1	80.0	Cry8Ab1	80.03	Cry8Ab1	80.27	Cry8Ab1	80.0
AMR88428.1	Cry1Fb4	99.8	Cry1Fb4	99.83	Cry1Fb4	99.82	Cry1Fb4	99.8
AMR88562.1	Cry1Hb1	100.0	Cry1Hb1	100.00	Cry1Hb1	100.00	Cry1Hb1	100.0
AMR88563.1	Cry1Bb1	99.9	Cry1Bb1	99.92	Cry1Bc1	100.00	Cry1Bb1	99.9
AMR88565.1	Cry1Ab18	100.0	Cry1Ab18	100.00	Cry1Ab18	100.00	Cry1Ab18	100.0
AMR88570.1	Cry1Nb1	100.0	–	–	Cry1Ca4	52.73	PesticidalProtein	100.0
AMR88574.1	Cry1Ja2	100.0	Cry1Ja1	99.57	Cry1Ja1	99.09	Cry1Ja1	99.6
AMR88576.1	Cry1Id1	100.0	Cry1Id1	100.00	Cry1Id1	100.00	Cry1Id1	100.0
AMR88578.1	Cry1Da3	99.9	Cry1Da2	96.82	Cry1Da2	99.82	Cry1Da1	96.8
AMR88604.1	Cry2Ad1	100.0	Cry2Ad1	100.00	*	*	Cry2Ad1	100.0
AND11132.1	Cry2Ab16	100.0	Cry2Ab3	100.00	*	*	Cry2Ab1	100.0
AND11141.1	Cry1Ae1	99.9	Cry1Ae1	99.92	Cry1Ae1	100.00	Cry1Ae1	99.9
ANS46158.1	Cry13Aa1	100.0	Cry13Aa1	100.00	Cry13Aa1	100.00	Cry13Aa1	100.0
ANS46780.1	Cry13Aa1	50.1	Cry13Aa1	51.82	*	*	Cry13Aa1	50.1
ANS50148.1	Cry13Aa1	87.1	Cry13Aa1	87.06	Cry13Aa1	100.00	Cry13Aa1	87.1
AOM14729.1	Cry21Ca2	37.1	Cry21Ba1	36.22	*	*	Cry21Ba1	34.5
OIX12409.1	Cry9Ea1	100.0	Cry9Ea2	100.00	Cry9Ea2	100.00	Cry9Ea1	100.0
OIX18506.1	Cry1Ca8	100.0	Cry1Ca9	100.00	Cry1Ca9	100.00	Cry1Ca7	100.0
OIX18511.1	Cry1Ia14	100.0	Cry1Ia2	100.00	Cry1Ia2	100.00	Cry1Ia10	100.0
OIX15228.1	Cry2Ab16	100.0	Cry2Ab3	100.00	*	*	Cry2Ab1	100.0
OIX23464.1	Cry1Ia14	100.0	Cry1Ia2	100.00	Cry1Ia2	100.00	Cry1Ia10	100.0
OIX23467.1	Cry2Aa1	100.0	Cry2Aa9	100.00	*	*	Cry2Aa1	100.0
OIX21407.1	Cry2Ab16	100.0	Cry2Ab3	100.00	*	*	Cry2Ab1	100.0
OMH24296.1	Cry8Pa2	100.0	Cry8Ba1	68.40	Cry8Ba1	65.58	Cry8Ba1	68.6
OMH23709.1	Cry8Qa1	100.0	Cry8Fa1	67.14	Cry8Fa1	63.50	Cry8Fa1	67.1
OMH24237.1	Cry8Kb1	100.0	Cry8Kb	100.00	Cry8Ka2	82.87	Cry8Kb	100.0
AQY42679.1	Cry1Ab34	100.0	–	–	Cry1Ab5	96.60	Cry1Ab18	95.4
AQY42680.1	Cry1Ia14	99.2	Cry1Ia2	99.17	Cry1Ia2	99.12	Cry1Ia10	99.2
AQY42687.1	Cry1Bd2	99.8	Cry1Bd2	99.84	Cry1Bd1	100.00	Cry1Bd2	99.8
ARP61632.1	Cry1Ba1	99.9	Cry1Ba5	99.92	Cry1Ba5	100.00	Cry1Ba1	99.9
ARP61819.1	Cry1Ab12	100.0	Cry1Ab9	100.00	Cry1Ab5	100.00	Cry1Ab1	100.0
OTW73736.1	Cry1Bd1	99.9	Cry1Bd1	99.92	Cry1Bd1	100.00	Cry1Bd1	99.9
OTW73398.1	Cry9Ba2	99.4	Cry9Ba−like	100.00	Cry9Ba2	100.00	Cry9Ba−like	100.0
OTW63083.1	Cry2Ab4	96.7	Cry2Ab4	96.68	*	*	Cry2Ab4	96.7
OTW61456.1	Cry2Ab4	98.6	Cry2Ab4	98.58	*	*	Cry2Ab4	98.6
OTW56025.1	Cry1Ga1	100.0	Cry1Ga1	100.00	Cry1Ga1	100.00	Cry1Ga1	100.0
OTW54288.1	Cry9Aa1	100.0	Cry9Aa1	100.00	Cry9Aa1	100.00	Cry9Aa1	100.0
OTW53899.1	Cry1Cb1	100.0	Cry1Cb2	100.00	Cry1Cb2	100.00	Cry1Cb2	100.0
OTW53896.1	Cry1Gb2	99.8	Cry1Gb2	99.83	Cry1Gb2	100.00	Cry1Gb2	99.8
OTW51791.1	Cry1Db1	100.0	Cry1Db2	100.00	Cry1Db2	100.00	Cry1Db1	100.0
OTW51792.1	Cry1Fb1	100.0	Cry1Fb5	100.00	Cry1Fb2	100.00	Cry1Fb1	100.0
OTW51788.1	Cry1Ab35	99.6	Cry1Ab18	98.94	Cry1Ab5	100.00	Cry1Ab18	98.9
OTW51776.1	Cry1Ib7	99.9	Cry1Ib3	99.86	Cry1Ib3	100.00	Cry1Ib3	99.9
OTW47541.1	Cry2Ac6	100.0	Cry2Ac6	100.00	*	*	Cry2Ac6	100.0
OTW46212.1	Cry7Ab4	99.8	Cry7Ab4	99.82	Cry7Ab4	100.00	Cry7Ab4	99.8
OTW42787.1	Cry1Ba1	99.9	Cry1Ba5	99.92	Cry1Ba5	100.00	Cry1Ba1	99.9
OTW70091.1	Cry21Fa1	31.0	Cry21Ba1	32.58	*	*	Cry5Ca2	34.0
OTW62068.1	Cry21Fa1	31.0	–	–	*	*	Cry5Ca2	34.0
OTW93738.1	Cry9Aa4	99.9	Cry9Aa1	97.99	Cry9Aa1	99.66	Cry9Aa1	98.0
OTW87134.1	Cry9Eb1	100.0	Cry9Eb1	100.00	Cry9Eb1	100.00	Cry9Eb1	100.0
OTW84856.1	Cry9Ee2	99.1	Cry9Ea−like	100.00	Cry9−like	72.55	Cry9Ea−like	100.0
OTW84781.1	Cry1Na1	100.0	Cry1B−like	100.00	Cry1Ca5	52.39	Cry1B−like	100.0
OTW84538.1	Cry9Da1	100.0	Cry9Eb−like	100.00	Cry9Da1	100.00	Cry9Da1	100.0
OTW81389.1	Cry1Ie2	100.0	–	–	Cry1Ie1	98.94	Cry1Ie1	96.0
OTX08732.1	Cry1Na1	100.0	Cry1B−like	100.00	Cry1Ca5	52.39	Cry1B−like	100.0
OTX08738.1	Cry9Aa4	99.8	Cry9Aa1	97.66	Cry9Aa1	99.66	Cry9Aa1	97.7

OTW94166.1	Cry9Ee2	98.9	Cry9Ea−like	99.02	Cry9−like	72.55	Cry9Ea−like	99.0
OTW94050.1	Cry9Da3	99.7	Cry9Eb−like	99.68	Cry9Da1	100.00	Cry9Eb−like	99.7
OTW90757.1	Cry1Ie2	100.0	−	−	Cry1Ie1	98.94	Cry1Ie1	96.0
OTW84525.1	Cry1Ie2	100.0	−	−	Cry1Ie1	98.94	Cry1Ie1	96.0
OTW84402.1	Cry1Na1	100.0	Cry1B−like	100.00	Cry1Ca5	52.39	Cry1B−like	100.0
OTW84346.1	Cry9Da4	99.7	Cry9Eb−like	100.00	Cry9Da1	100.00	Cry9Eb−like	100.0
OTW81421.1	Cry9Eb3	95.6	Cry9Eb1	94.97	Cry9Eb1	100.00	Cry9Eb1	95.0
OTX02121.1	Cry7Ab4	99.8	Cry7Ab4	99.82	Cry7Ab4	100.00	Cry7Ab4	99.8
OTW70710.1	Cry7Ba1	100.0	Cry7Ba1	100.00	Cry7Ba1	100.00	Cry7Ba1	100.0
OTW70715.1	Cry7Ia1	100.0	Cry7Ba1−like	99.91	Cry7Ba1	44.29	Cry7Ba1−like	99.9
OTX23942.1	Cry1Ie2	100.0	−	−	Cry1Ie1	98.94	Cry1Ie1	96.0
OTX23531.1	Cry9Da4	99.7	Cry9Eb−like	100.00	Cry9Da1	100.00	Cry9Eb−like	100.0
OTX21375.1	Cry1Na1	100.0	Cry1B−like	100.00	Cry1Ca5	52.39	Cry1B−like	100.0
OTX17826.1	Cry9Eb3	95.6	−	−	Cry9Eb1	100.00	Cry9Eb1	95.0
OTX40985.1	Cry1Ac2	99.9	Cry1Ac2	99.92	Cry1Ac2	100.00	Cry1Ac2	99.9
OTX38902.1	Cry2Ab4	100.0	Cry2Ab4	100.00	*	*	Cry2Ab4	100.0
OTX17809.1	Cry1Ia14	99.6	Cry1Ia2	99.58	Cry1Ia2	99.47	Cry1Ia10	99.6
OTX17812.1	Cry2Aa1	100.0	Cry2Aa9	100.00	*	*	Cry2Aa1	100.0
OTX56884.1	Cry7Ba1	100.0	Cry7Ba1	100.00	Cry7Ba1	100.00	Cry7Ba1	100.0
OTX56889.1	Cry7Ia1	100.0	Cry7Ba1−like	99.91	Cry7Ba1	44.29	Cry7Ba1−like	99.9
OTX78579.1	Cry4Aa2	100.0	Cry4Aa4	100.00	Cry4Aa4	100.00	Cry4Aa2	100.0
OTX77086.1	Cry10Aa3	100.0	Cry10Aa4	100.00	Cry10Aa4	100.00	Cry10Aa3	100.0
OTX69600.1	Cry4Ba1	100.0	−	−	cry4Ba	100.00	Cry4Ba	100.0
OTX69582.1	Cry11Aa1	100.0	Cry11Aa3	100.00	*	*	Cry11Aa1	100.0
OTX77050.1	Cry28Aa2	99.7	Cry28Aa2	99.73	Cry28Aa2	99.47	Cry28Aa2	99.7
OTX75968.1	Cry26Aa1	95.5	−	−	Cry26Aa1	100.00	Cry26Aa1	95.5
OTX71618.1	Cry28Aa2	99.7	Cry28Aa2	99.73	Cry28Aa2	99.47	Cry28Aa2	99.7
OTX71525.1	Cry28Aa2	99.7	Cry28Aa2	99.73	Cry28Aa2	99.47	Cry28Aa2	99.7
OTX71494.1	Cry7Ba1	100.0	Cry7Ba1	100.00	Cry7Ba1	100.00	Cry7Ba1	100.0
OTX71499.1	Cry7Ia1	100.0	Cry7Ba1−like	99.91	Cry7Ba1	44.29	Cry7Ba1−like	99.9
OTY10661.1	Cry7Ka1	100.0	Cry7Ab4	67.84	Cry7Ab4	54.27	Cry7Ab4	67.8
OTY00887.1	Cry7Cb1	60.4	Cry7Ba1	57.07	Cry8Db1	31.97	Cry7Ba1	57.2
OTY00858.1	Cry7Gc1	99.8	Cry7Ba1	59.77	Cry61Aa3	43.06	Cry7Ba1	59.8
OTY42695.1	Cry3Aa1	100.0	Cry3Aa7	100.00	Cry3Aa7	100.00	Cry3Aa1	100.0
OTY31851.1	Cry28Aa2	99.7	Cry28Aa2	99.73	Cry28Aa2	99.47	Cry28Aa2	99.7
OTY27002.1	Cry7Ba1	100.0	Cry7Ba1	100.00	Cry7Ba1	100.00	Cry7Ba1	100.0
OTY27007.1	Cry7Ia1	100.0	Cry7Ba1−like	99.91	Cry7Ba1	44.29	Cry7Ba1−like	99.9
OTY48305.1	Cry8Ac1	100.0	Cry8Ab1−like	100.00	Cry8Ab1	74.96	Cry8Ab1−like	100.0
OTY48272.1	Cry8Ac1	100.0	Cry8Ab1−like	100.00	Cry8Ab1	74.96	Cry8Ab1−like	100.0
OTY48296.1	Cry8Ac1	100.0	Cry8Ab1−like	100.00	Cry8Ab1	74.96	Cry8Ab1−like	100.0
OTY42322.1	Cry8Ac1	100.0	Cry8Ab1−like	100.00	Cry8Ab1	74.96	Cry8Ab1−like	100.0
OTY44027.1	Cry2Ab16	100.0	Cry2Ab3	100.00	*	*	Cry2Ab1	100.0
OTY60291.1	Cry21Da1	100.0	Cry21Ba2−like	100.00	*	*	Cry21Ba2−like	100.0
OTY57628.1	Cry21Fa1	47.3	Cry21Ba2−like	47.95	*	*	Cry21Ba2−like	46.7
OTY55646.1	Cry21Ca2	100.0	Cry21Ba1	52.90	*	*	Cry21Ba1	53.2
OTY74981.1	Cry8Ta1	100.0	Cry8Ia1	59.98	Cry8Da3	50.42	Cry8Da2	58.8
OTY74274.1	Cry8Ma3	100.0	Cry8−like	100.00	Cry8Ma2	100.00	Cry8−like	100.0
OTY74147.1	Cry8Ia4	100.0	Cry8Ia1	99.50	Cry8Ia1	99.13	Cry8Ia1	99.5
OTY85625.1	Cry1Ba2	99.9	Cry1Ba4	99.92	Cry1Ba4	100.00	Cry1Ba2	99.9
OTX53140.1	Cry7Ia1	100.0	Cry7Ba1−like	99.91	Cry7Ba1	44.29	Cry7Ba1−like	99.9
OTX53145.1	Cry7Ba1	100.0	Cry7Ba1	100.00	Cry7Ba1	100.00	Cry7Ba1	100.0
OTX96001.1	Cry8Pa2	100.0	Cry8Ba1	71.20	Cry8Ba1	65.58	Cry8Ba1	71.2
OTX94785.1	Cry8Qa1	100.0	Cry8Fa1	69.83	Cry8Fa1	63.50	Cry8Fa1	69.8
OTX85968.1	Cry8Kb1	100.0	Cry8Kb	100.00	Cry8Ka2	82.87	Cry8Kb	100.0
OTY77561.1	Cry1Bd1	99.9	Cry1Bd1	99.92	Cry1Bd1	100.00	Cry1Bd1	99.9
OTY74986.1	Cry1Ib7	99.9	Cry1Ib3	99.86	Cry1Ib3	100.00	Cry1Ib3	99.9
OTY64265.1	Cry2Ac6	100.0	Cry2Ac6	100.00	*	*	Cry2Ac6	100.0
OTY63536.1	Cry1Db1	100.0	Cry1Db2	100.00	Cry1Db2	100.00	Cry1Db1	100.0

OTY63537.1	Cry1Fb1	100.0	Cry1Fb5	100.00	Cry1Fb2	100.00	Cry1Fb1	100.0
OTY56302.1	Cry9Ba2	99.6	Cry9Ba−like	99.82	Cry9Ba2	100.00	Cry9Ba−like	99.8
OTY56301.1	Cry9Aa1	99.8	Cry9Aa1	99.83	Cry9Aa1	100.00	Cry9Aa1	99.8
OTY56195.1	Cry1Gb2	99.9	Cry1Gb2	99.91	Cry1Gb2	100.00	Cry1Gb2	99.9
OTY54786.1	Cry1Ab35	100.0	Cry1Ab18	99.11	Cry1Ab5	100.00	Cry1Ab18	99.1
OTY50446.1	Cry2Ab4	98.6	Cry2Ab4	98.58	*	*	Cry2Ab4	98.6
OTY50443.1	Cry1Cb1	100.0	Cry1Cb2	100.00	Cry1Cb2	100.00	Cry1Cb2	100.0
OTY50440.1	Cry2Ab4	96.7	Cry2Ab4	96.68	*	*	Cry2Ab4	96.7
OTY50433.1	Cry1Ga1	100.0	Cry1Ga1	100.00	Cry1Ga1	100.00	Cry1Ga1	100.0
OTY90981.1	Cry1Ab35	99.9	Cry1Ab18	97.63	Cry1Ab5	100.00	Cry1Ab18	97.3
OTY88175.1	Cry1Bd1	99.9	Cry1Bd1	99.92	Cry1Bd1	100.00	Cry1Bd1	99.9
OTY85311.1	Cry1Ga1	100.0	Cry1Ga1	100.00	Cry1Ga1	100.00	Cry1Ga1	100.0
OTY84534.1	Cry1Gb2	99.9	Cry1Gb2	99.91	Cry1Gb2	100.00	Cry1Gb2	99.9
OTY81209.1	Cry9Ba2	99.9	Cry9Ba−like	100.00	Cry9Ba2	100.00	Cry9Ba−like	100.0
OTY81129.1	Cry1Ib7	99.9	Cry1Ib3	99.86	Cry1Ib3	100.00	Cry1Ib3	99.9
OTY81130.1	Cry1Db1	100.0	Cry1Db1	100.00	Cry1Db2	100.00	Cry1Db1	100.0
OTY81131.1	Cry1Fb1	100.0	Cry1Fb5	100.00	Cry1Fb2	100.00	Cry1Fb1	100.0
OTY81124.1	Cry9Aa1	100.0	Cry9Aa1	100.00	Cry9Aa1	100.00	Cry9Aa1	100.0
OTY81076.1	Cry2Ac6	100.0	Cry2Ac6	100.00	*	*	Cry2Ac6	100.0
OTY80983.1	Cry1Cb1	100.0	Cry1Cb2	100.00	Cry1Cb2	100.00	Cry1Cb2	100.0
OTY80916.1	Cry2Ab4	100.0	Cry2Ab4	100.00	*	*	Cry2Ab4	100.0
OTZ05470.1	Cry2Ab16	100.0	Cry2Ab3	100.00	*	*	Cry2Ab1	100.0
OTZ05476.1	Cry1Aa12	100.0	Cry1Aa3	100.00	Cry1Aa3	100.00	Cry1Aa12	100.0
OTZ05477.1	Cry1Ia14	100.0	Cry1Ia2	100.00	Cry1Ia2	100.00	Cry1Ia10	100.0
OTZ00150.1	Cry1Da1	100.0	Cry1Da2	100.00	Cry1Da2	100.00	Cry1Da1	100.0
OTZ00152.1	Cry1Ca8	100.0	Cry1Ca9	100.00	Cry1Ca9	100.00	Cry1Ca7	100.0
OTY84077.1	Cry1Ia14	100.0	Cry1Ia2	100.00	Cry1Ia2	100.00	Cry1Ia10	100.0
OTY83992.1	Cry2Ab16	100.0	Cry2Ab3	100.00	*	*	Cry2Ab1	100.0
OTY76348.1	Cry1Ca8	100.0	Cry1Ca9	100.00	Cry1Ca9	100.00	Cry1Ca7	100.0
OTY76350.1	Cry1Da1	100.0	Cry1Da2	100.00	Cry1Da2	100.00	Cry1Da1	100.0
OTZ15274.1	Cry1Ca8	100.0	Cry1Ca9	100.00	Cry1Ca9	100.00	Cry1Ca7	100.0
OTZ15276.1	Cry1Da1	100.0	Cry1Da2	100.00	Cry1Da2	100.00	Cry1Da1	100.0
OTZ15087.1	Cry1Ia14	100.0	Cry1Ia2	100.00	Cry1Ia2	100.00	Cry1Ia10	100.0
OTZ13168.1	Cry2Ab16	100.0	Cry2Ab3	100.00	*	*	Cry2Ab1	100.0
OTZ11691.1	Cry9Ea1	100.0	Cry9Ea2	100.00	Cry9Ea2	100.00	Cry9Ea1	100.0
OTY98192.1	Cry1Ia14	100.0	Cry1Ia2	100.00	Cry1Ia2	100.00	Cry1Ia10	100.0
OTY98193.1	Cry1Aa12	100.0	Cry1Aa3	100.00	Cry1Aa3	100.00	Cry1Aa12	100.0
OTY98199.1	Cry2Ab16	100.0	Cry2Ab3	100.00	*	*	Cry2Ab1	100.0
OTY98186.1	Cry9Ea1	100.0	Cry9Ea2	100.00	Cry9Ea2	100.00	Cry9Ea1	100.0
OTZ48089.1	Cry1Ac5	100.0	Cry1Ac5	100.00	Cry1Ac32	100.00	Cry1Ac17	100.0
OTZ43079.1	Cry2Ab16	100.0	Cry2Ab3	100.00	*	*	Cry2Ab1	100.0
OTZ38706.1	Cry1Aa11	100.0	Cry1Aa8	100.00	Cry1Aa5	100.00	Cry1Aa11	100.0
OTZ20302.1	Cry1Ia14	100.0	Cry1Ia2	100.00	Cry1Ia2	100.00	Cry1Ia10	100.0
OTZ20305.1	Cry2Aa1	100.0	Cry2Aa9	100.00	*	*	Cry2Aa1	100.0
OTZ18389.1	Cry1Ea11	100.0	Cry1Ea7	100.00	Cry1Ea5	100.00	Cry1Ea11	100.0
OTZ15900.1	Cry2Aa1	100.0	Cry2Aa9	100.00	*	*	Cry2Aa1	100.0
OTZ15903.1	Cry1Ia14	99.6	Cry1Ia2	99.58	Cry1Ia2	99.47	Cry1Ia10	99.6
OTZ14059.1	Cry1Ac2	99.8	Cry1Ac2	99.83	Cry1Ac2	100.00	Cry1Ac2	99.8
OTZ58098.1	Cry8Aa1	46.1	Cry8Aa1	45.93	Cry8Aa1	47.00	Cry8Aa1	46.1
OTZ54220.1	Cry2Ad1	100.0	Cry2Ad1	100.00	*	*	Cry2Ad1	100.0
OTZ53847.1	Cry1Hb1	100.0	Cry1Hb1	100.00	Cry1Hb1	100.00	Cry1Hb1	100.0
OTZ53849.1	Cry1Bb1	99.9	Cry1Bb1	99.92	Cry1Bc1	100.00	Cry1Bb1	99.9
OTZ53852.1	Cry1Ab18	100.0	Cry1Ab18	100.00	Cry1Ab18	100.00	Cry1Ab18	100.0
OTZ53859.1	Cry1Nb1	100.0	–	–	Cry1Ca4	52.73	PesticidalProtein	100.0
OTZ53864.1	Cry1Ja2	100.0	Cry1Ja1	99.57	Cry1Ja1	99.09	Cry1Ja1	99.6
OTZ53867.1	Cry1Id1	100.0	Cry1Id1	100.00	Cry1Id1	100.00	Cry1Id1	100.0
OTZ53868.1	Cry1Da3	99.9	Cry1Da2	96.82	Cry1Da2	99.82	Cry1Da1	96.8
OTZ39194.1	Cry1Fb4	99.8	Cry1Fb4	99.83	Cry1Fb4	99.82	Cry1Fb4	99.8

OTZ32741.1	Cry1Ka2	99.8	Cry1Ka1	98.99	Cry1Ka1	98.58	Cry1Ka1	98.2
OTZ30763.1	Cry2Ad1	100.0	Cry2Ad1	100.00	*	*	Cry2Ad1	100.0
OTZ30108.1	Cry1Hb1	100.0	Cry1Hb1	100.00	Cry1Hb1	100.00	Cry1Hb1	100.0
OTZ30110.1	Cry1Bb1	99.9	Cry1Bb1	99.92	Cry1Bc1	100.00	Cry1Bb1	99.9
OTZ30113.1	Cry1Ab18	100.0	Cry1Ab18	100.00	Cry1Ab18	100.00	Cry1Ab18	100.0
OTZ30120.1	Cry1Ib11	99.9	Cry1Ib9	98.75	Cry1Ib6	100.00	Cry1Ib10	98.7
OTZ63029.1	Cry1Gb2	99.8	Cry1Gb2	99.83	Cry1Gb2	100.00	Cry1Gb2	99.8
OTZ53632.1	Cry1Bd1	99.9	Cry1Bd1	99.92	Cry1Bd1	100.00	Cry1Bd1	99.9
OTZ53086.1	Cry2Ab4	100.0	Cry2Ab4	100.00	*	*	Cry2Ab4	100.0
OTZ52987.1	Cry1Cb1	100.0	Cry1Cb2	100.00	Cry1Cb2	100.00	Cry1Cb2	100.0
OTZ48347.1	Cry1Ab35	99.6	Cry1Ab18	98.94	Cry1Ab5	100.00	Cry1Ab18	98.9
OTZ47101.1	Cry9Aa1	99.5	Cry9Aa1	99.48	Cry9Aa1	100.00	Cry9Aa1	99.5
OTZ46996.1	Cry9Ba2	99.9	Cry9Ba−like	100.00	Cry9Ba2	100.00	Cry9Ba−like	100.0
OTZ46390.1	Cry1Ga1	100.0	Cry1Ga1	100.00	Cry1Ga1	100.00	Cry1Ga1	100.0
OTZ46143.1	Cry2Ab4	98.1	Cry2Ab4	98.10	*	*	Cry2Ab4	98.1
OTZ43629.1	Cry1Db1	100.0	Cry1Db2	100.00	Cry1Db2	100.00	Cry1Db1	100.0
OTZ43630.1	Cry1Fb1	100.0	Cry1Fb5	100.00	Cry1Fb2	100.00	Cry1Fb1	100.0
OTZ43567.1	Cry1Ib7	99.9	Cry1Ib3	99.86	Cry1Ib3	100.00	Cry1Ib3	99.9
OTZ43161.1	Cry2Ac6	100.0	Cry2Ac6	100.00	*	*	Cry2Ac6	100.0
OTZ77559.1	Cry7Ab3	99.8	Cry7Ab8	99.82	Cry7Ab8	99.64	Cry7Ab3	99.8
OUA04284.1	Cry2Ab16	100.0	Cry2Ab3	100.00	*	*	Cry2Ab1	100.0
OUA02994.1	Cry1Ia14	100.0	Cry1Ia2	100.00	Cry1Ia2	100.00	Cry1Ia10	100.0
OTZ94741.1	Cry1Ac10	100.0	Cry1Ac9	100.00	Cry1Ac9	100.00	Cry1Ac1	100.0
OTZ93984.1	Cry1Aa12	100.0	Cry1Aa3	100.00	Cry1Aa3	100.00	Cry1Aa12	100.0
OTZ98517.1	Cry1Ea11	100.0	Cry1Ea7	100.00	Cry1Ea5	100.00	Cry1Ea11	100.0
OUA02427.1	Cry1Ea11	100.0	Cry1Ea7	100.00	Cry1Ea5	100.00	Cry1Ea11	100.0
OTZ97385.1	Cry1Ac2	99.8	Cry1Ac2	99.83	Cry1Ac2	100.00	Cry1Ac2	99.8
OTZ92959.1	Cry1Ia14	99.6	Cry1Ia2	99.58	Cry1Ia2	99.47	Cry1Ia10	99.6
OTZ92962.1	Cry2Aa1	100.0	Cry2Aa9	100.00	*	*	Cry2Aa1	100.0
OUA19853.1	Cry9Ea1	100.0	Cry9Ea2	100.00	Cry9Ea2	100.00	Cry9Ea1	100.0
OUA19157.1	Cry2Ab16	100.0	Cry2Ab3	100.00	*	*	Cry2Ab1	100.0
OUA19085.1	Cry1Ia14	100.0	Cry1Ia2	100.00	Cry1Ia2	100.00	Cry1Ia10	100.0
OUA13599.1	Cry1Ca8	100.0	Cry1Ca9	100.00	Cry1Ca9	100.00	Cry1Ca7	100.0
OUA13601.1	Cry1Da1	100.0	Cry1Da2	100.00	Cry1Da2	100.00	Cry1Da1	100.0
OUA29863.1	Cry9Ea1	100.0	Cry9Ea2	100.00	Cry9Ea2	100.00	Cry9Ea1	100.0
OUA23293.1	Cry1Ac10	100.0	Cry1Ac9	100.00	Cry1Ac9	100.00	Cry1Ac1	100.0
OUA22636.1	Cry1Aa12	100.0	Cry1Aa3	100.00	Cry1Aa3	100.00	Cry1Aa12	100.0
OUA22575.1	Cry1Ia14	100.0	Cry1Ia2	100.00	Cry1Ia2	100.00	Cry1Ia10	100.0
OUA22580.1	Cry1Ca8	100.0	Cry1Ca9	100.00	Cry1Ca9	100.00	Cry1Ca7	100.0
OUA22582.1	Cry1Da1	99.6	Cry1Da2	99.57	Cry1Da2	99.08	Cry1Da1	99.6
OUA14469.1	Cry2Ab16	100.0	Cry2Ab3	100.00	*	*	Cry2Ab1	100.0
OUA21361.1	Cry9Ea1	100.0	Cry9Ea2	100.00	Cry9Ea2	100.00	Cry9Ea1	100.0
OUA20993.1	Cry2Ab16	100.0	Cry2Ab3	100.00	*	*	Cry2Ab1	100.0
OUA20999.1	Cry1Aa12	100.0	Cry1Aa3	100.00	Cry1Aa3	100.00	Cry1Aa12	100.0
OUA21000.1	Cry1Ia14	100.0	Cry1Ia2	100.00	Cry1Ia2	100.00	Cry1Ia10	100.0
OUA21005.1	Cry1Ca8	100.0	Cry1Ca9	100.00	Cry1Ca9	100.00	Cry1Ca7	100.0
OUA21007.1	Cry1Da1	100.0	Cry1Da2	100.00	Cry1Da2	100.00	Cry1Da1	100.0
OUA49687.1	Cry2Ab16	100.0	Cry2Ab3	100.00	*	*	Cry2Ab1	100.0
OUA40858.1	Cry1Ab12	100.0	Cry1Ab20	100.00	Cry1Ab5	100.00	Cry1Ab1	100.0
OUA40850.1	Cry1Aa12	99.9	Cry1Aa3	99.87	Cry1Aa3	100.00	Cry1Aa12	99.9
OUA34007.1	Cry1Ia14	100.0	Cry1Ia2	100.00	Cry1Ia2	100.00	Cry1Ia10	100.0
OUA34012.1	Cry1Ca8	100.0	Cry1Ca9	100.00	Cry1Ca9	100.00	Cry1Ca7	100.0
OUA34014.1	Cry1Da1	100.0	Cry1Da2	100.00	Cry1Da2	100.00	Cry1Da1	100.0
OUA38461.1	Cry9Ea1	100.0	Cry9Ea2	100.00	Cry9Ea2	100.00	Cry9Ea1	100.0
OUA36885.1	Cry1Ia14	100.0	Cry1Ia2	100.00	Cry1Ia2	100.00	Cry1Ia10	100.0
OUA36886.1	Cry1Aa12	100.0	Cry1Aa3	100.00	Cry1Aa3	100.00	Cry1Aa12	100.0
OUA36892.1	Cry2Ab16	100.0	Cry2Ab3	100.00	*	*	Cry2Ab1	100.0
OUA45353.1	Cry9Ea1	100.0	Cry9Ea2	100.00	Cry9Ea2	100.00	Cry9Ea1	100.0

OUA40248.1	Cry1Ab12	100.0	Cry1Ab9	100.00	Cry1Ab5	100.00	Cry1Ab1	100.0
OUA35577.1	Cry1Aa12	99.9	Cry1Aa3	99.87	Cry1Aa3	100.00	Cry1Aa12	99.9
OUA34148.1	Cry2Ab16	100.0	Cry2Ab3	100.00	*	*	Cry2Ab1	100.0
OUA32933.1	Cry1Ia14	100.0	Cry1Ia2	100.00	Cry1Ia2	100.00	Cry1Ia10	100.0
OUA32938.1	Cry1Ca8	100.0	Cry1Ca9	100.00	Cry1Ca9	100.00	Cry1Ca7	100.0
OUA32940.1	Cry1Da1	100.0	Cry1Da2	100.00	Cry1Da2	100.00	Cry1Da1	100.0
OUA82733.1	Cry9Ea1	100.0	Cry9Ea2	100.00	Cry9Ea2	100.00	Cry9Ea1	100.0
OUA67399.1	Cry2Ab16	100.0	Cry2Ab3	100.00	*	*	Cry2Ab1	100.0
OUA67405.1	Cry1Aa12	100.0	Cry1Aa3	100.00	Cry1Aa3	100.00	Cry1Aa12	100.0
OUA67406.1	Cry1Ia14	100.0	Cry1Ia2	100.00	Cry1Ia2	100.00	Cry1Ia10	100.0
OUA67411.1	Cry1Ca8	100.0	Cry1Ca9	100.00	Cry1Ca9	100.00	Cry1Ca7	100.0
OUA67413.1	Cry1Da1	100.0	Cry1Da2	100.00	Cry1Da2	100.00	Cry1Da1	100.0
OUA77719.1	Cry9Ea1	100.0	Cry9Ea2	100.00	Cry9Ea2	100.00	Cry9Ea1	100.0
OUA76906.1	Cry2Ab16	100.0	Cry2Ab3	100.00	*	*	Cry2Ab1	100.0
OUA76864.1	Cry1Da1	100.0	Cry1Da2	100.00	Cry1Da2	100.00	Cry1Da1	100.0
OUA76866.1	Cry1Ca8	100.0	Cry1Ca9	100.00	Cry1Ca9	100.00	Cry1Ca7	100.0
OUA76871.1	Cry1Ia14	100.0	Cry1Ia2	100.00	Cry1Ia2	100.00	Cry1Ia10	100.0
OUB04094.1	Cry41Ba2	100.0	Cry41Ba2	100.00	Cry41Ba2	100.00	Cry41Ba2	100.0
OUA92623.1	Cry41Ba1	100.0	Cry66Aa2	100.00	Cry66Aa2	100.00	Cry66Aa2	100.0
OUB62847.1	Cry20Ba2	100.0	Cry20Ba1	98.95	Cry20Ba1	98.73	Cry20Ba1	98.8
OUB51426.1	Cry19Ba1	100.0	Cry19Ba1	99.85	Cry19Ba1	100.00	Cry19Ba1	100.0
OUB36504.1	Cry73Aa1	100.0	Cry41Aa1−like	100.00	Cry32Da1	40.72	Cry41Aa1−like	100.0
OUB35201.1	Cry8Ba1	97.0	Cry8Ba1	98.37	Cry8Ba1	99.13	Cry8Ba1	97.0
OUB29323.1	Cry8Ba1	97.0	Cry8Ba1	98.37	Cry8Ba1	99.13	Cry8Ba1	97.0
OUB22932.1	Cry8Ba1	97.0	Cry8Ba1	98.37	Cry8Ba1	99.13	Cry8Ba1	97.0
OUB21150.1	Cry8Ba1	97.0	Cry8Ba1	98.37	Cry8Ba1	99.13	Cry8Ba1	97.0
OUB35623.1	Cry4Ba1	63.8	Cry4Ba5	63.88	cry4Ba	63.51	Cry4Ba	63.8
OUB78400.1	Cry25Aa1	97.8	Cry25Aa1	97.78	Cry25Aa1	97.75	Cry25Aa1	97.8
OUB77820.1	Cry11Ba1	100.0	Cry11Ba1	100.00	*	*	Cry11Ba1	100.0
OUB77817.1	Cry70Bb1	67.1	Cry31Aa6−like	100.00	*	*	Cry31Aa6−like	100.0
OUC03368.1	Cry11Bb1	99.7	−	−	*	*	Cry11Bb1	99.7
OUC03129.1	Cry29Aa1	96.4	Cry29Aa1	96.43	*	*	Cry29Aa1	96.4
ARV91367.1	Cry7Ba1	100.0	Cry7Ba1	100.00	Cry7Ba1	100.00	Cry7Ba1	100.0
ARV91400.1	Cry7Ia1	100.0	Cry7Ba1−like	99.91	Cry7Ba1	44.29	Cry7Ba1−like	99.9
OXR51181.1	Cry9Ea1	100.0	Cry9Ea2	100.00	Cry9Ea2	100.00	Cry9Ea1	100.0
OXR53711.1	Cry1Ia14	100.0	Cry1Ia2	100.00	Cry1Ia2	100.00	Cry1Ia10	100.0
OXR53716.1	Cry1Ca8	100.0	Cry1Ca9	100.00	Cry1Ca9	100.00	Cry1Ca7	100.0
OXR53718.1	Cry1Da1	100.0	Cry1Da2	100.00	Cry1Da2	100.00	Cry1Da1	100.0
OXR51828.1	Cry2Ab16	100.0	Cry2Ab3	100.00	*	*	Cry2Ab1	100.0
AST05277.1	Cry1Ba1	100.0	Cry1Ba5	100.00	Cry1Ba5	100.00	Cry1Ba1	100.0
PHQ17955.1	Cry1Ca8	100.0	Cry1Ca9	100.00	Cry1Ca9	100.00	Cry1Ca7	100.0
PHQ17551.1	Cry1Ia14	100.0	Cry1Ia2	100.00	Cry1Ia2	100.00	Cry1Ia10	100.0
PHQ17458.1	Cry9Ea1	100.0	Cry9Ea2	100.00	Cry9Ea2	100.00	Cry9Ea1	100.0
PHQ17345.1	Cry1Da1	100.0	Cry1Da2	100.00	Cry1Da2	100.00	Cry1Da1	100.0
PHQ18226.1	Cry2Ab16	100.0	Cry2Ab3	100.00	*	*	Cry2Ab1	100.0
PDY40352.1	Cry5Ba1	99.8	Cry5Ba1	99.84	*	*	Cry5Ba1	99.8
PDY37670.1	Cry65Aa2	55.6	Cry65Aa2	55.54	*	*	Cry65Aa2	55.6
PEA88281.1	Cry19Aa1	29.2	Cry27Aa1	30.84	Cry27Aa1	31.53	Cry19Aa1	29.2
PEA91147.1	Cry43Cb1	43.9	Cry53Aa1	39.34	Cry43Aa2	38.80	Cry43Aa2	37.3
PEA86378.1	Cry4Cb3	34.4	Cry4Cb2	34.44	Cry4Cb2	34.59	Cry4Cb2	34.3
PEE63029.1	Cry32Wa1	39.0	Cry41Aa1−like	38.02	Cry41Ab1	35.12	Cry41Ab1	33.8
PEE68563.1	Cry8Ja1	60.3	Cry8Ja1	60.18	Cry8Ja1	62.52	Cry8Ja1	60.3
PEE85821.1	Cry32Wa1	39.0	−	−	Cry41Ab1	35.12	Cry41Ab1	33.8
PEK85070.1	Cry1Ia14	100.0	Cry1Ia2	100.00	Cry1Ia2	100.00	Cry1Ia10	100.0
PEK85073.1	Cry2Aa1	100.0	Cry2Aa9	100.00	*	*	Cry2Aa1	100.0
PEK97620.1	Cry2Ab16	100.0	Cry2Ab3	100.00	*	*	Cry2Ab1	100.0
PEU83535.1	Cry69Ab1	58.0	−	−	Cry53Ab1	35.63	Cry43Ba1	41.1
PEU81077.1	Cry9Ga1	57.2	Cry9Aa1	56.65	Cry9Aa1	57.92	Cry9Aa1	56.7

PEU80288.1	Cry56Aa4	56.4	Cry56Aa2	56.78	Cry56Aa1	55.70	Cry56Aa2	56.3
PEU78339.1	Cry40Da1	66.9	Cry40Da1	66.87	Cry40Da1	67.32	Cry40Da1	66.9
PEU78325.1	Cry71Aa1	65.9	Cry53Ab1	40.03	Cry53Ab1	40.44	Cry53Ab1	40.0
PEU74801.1	Cry70Ba1	97.3	Cry31Aa6−like	66.34	*	*	Cry31Aa6−like	65.1
PEU91245.1	Cry68Aa1	80.1	Cry9Aa1	34.33	Cry9Aa2	35.88	Cry9Aa1	34.0
PER42111.1	Cry69Ab1	58.0	−	−	Cry53Ab1	35.63	Cry43Ba1	41.1
PER41598.1	Cry71Aa1	65.9	−	−	Cry53Ab1	40.44	Cry53Ab1	40.0
PER39584.1	Cry9Ga1	57.2	−	−	Cry9Aa1	57.92	Cry9Aa1	56.7
PER38368.1	Cry68Aa1	80.1	−	−	Cry9Aa2	35.88	Cry9Aa1	34.0
PER37538.1	Cry56Aa4	56.4	−	−	Cry56Aa1	55.70	Cry56Aa2	56.3
PER55269.1	Cry40Da1	66.9	−	−	Cry40Da1	67.32	Cry40Da1	66.9
PER34669.1	Cry70Ba1	97.3	−	−	*	*	Cry31Aa6−like	65.1
PFB88478.1	Cry32Wa1	39.2	Cry41Aa1−like	38.17	Cry41Ab1	35.28	Cry41Ab1	33.8
PFE13212.1	Cry32Ma1	37.1	Cry41Aa1−like	38.17	Cry41Ab1	35.12	Cry41Ab1	33.8
PFE36768.1	Cry65Aa2	70.8	Cry65Aa2	70.66	*	*	Cry65Aa2	70.8
PFE29821.1	Cry5Ba1	99.8	Cry5Ba1	99.84	*	*	Cry5Ba1	99.8
PFE45262.1	Cry5Aa1	100.0	Cry5Aa1	100.00	*	*	Cry5Aa1	100.0
PFA82393.1	Cry1Ib11	99.9	Cry1Ib9	98.75	Cry1Ib6	100.00	Cry1Ib10	98.7
PFA97486.1	Cry1Ja1	100.0	Cry1Ja1	100.00	Cry1Ja1	100.00	Cry1Ja1	100.0
PEZ39117.1	Cry21Ba1	28.9	Cry21Ba1	28.31	*	*	Cry21Ba1	28.9
PFF41055.1	Cry8Aa1	46.1	−	−	Cry8Aa1	47.00	Cry8Aa1	46.1
PFI05053.1	Cry9Ga1	57.2	−	−	Cry9Aa1	57.92	Cry9Aa1	56.7
PFI04008.1	Cry71Aa1	65.9	−	−	Cry53Ab1	40.44	Cry53Ab1	40.0
PFH99743.1	Cry68Aa1	80.1	−	−	Cry9Aa2	35.88	Cry9Aa1	34.0
PFH99578.1	Cry56Aa4	56.4	−	−	Cry56Aa1	55.70	Cry56Aa2	56.3
PFH99374.1	Cry69Ab1	58.0	−	−	Cry53Ab1	35.63	Cry43Ba1	41.1
PFH98146.1	Cry40Da1	66.9	−	−	Cry40Da1	67.32	Cry40Da1	66.9
PFI06834.1	Cry70Ba1	97.3	−	−	*	*	Cry31Aa6−like	65.1
PFJ62899.1	Cry32Ma1	37.1	−	−	Cry41Ab1	35.12	Cry41Ab1	33.8
PFK99360.1	Cry32Wa1	39.2	Cry41Aa1−like	38.17	Cry41Ab1	35.61	Cry41Ab1	34.0
PFM86094.1	Cry32Wa1	39.0	−	−	Cry41Ab1	35.12	Cry41Ab1	33.8
PFP10217.1	Cry32Wa1	39.0	Cry41Aa1−like	38.02	Cry41Ab1	34.95	Cry41Ab1	33.7
PFT09171.1	Cry8Aa1	46.1	−	−	Cry8Aa1	47.00	Cry8Aa1	46.1
PFV01225.1	Cry32Ma1	37.1	−	−	Cry41Ab1	35.12	Cry41Ab1	33.8
PFT97155.1	Cry5Ba1	99.8	Cry5Ba1	99.84	*	*	Cry5Ba1	99.8
PFU07373.1	Cry65Aa2	70.8	−	−	*	*	Cry65Aa2	70.8
PFV85565.1	Cry32Wa1	39.2	Cry41Aa1−like	38.17	Cry41Ab1	35.28	Cry41Ab1	34.0
PFW25264.1	Cry71Aa1	65.9	−	−	Cry53Ab1	40.44	Cry53Ab1	40.0
PFW49142.1	Cry9Ga1	58.2	Cry9Aa2	57.92	Cry9Aa1	57.92	Cry9Aa1	57.8
PFW24146.1	Cry68Aa1	80.1	−	−	Cry9Aa2	35.88	Cry9Aa1	34.0
PFW19349.1	Cry69Ab1	58.0	−	−	Cry53Ab1	35.63	Cry43Ba1	41.1
PFW18565.1	Cry70Ba1	97.3	−	−	*	*	Cry31Aa6−like	65.1
PFW17822.1	Cry40Da1	66.9	−	−	Cry40Da1	67.32	Cry40Da1	66.9
PFW20907.1	Cry56Aa4	56.4	−	−	Cry56Aa1	55.70	Cry56Aa2	56.3
PGE54611.1	Cry2Aa1	100.0	Cry2Aa9	100.00	*	*	Cry2Aa1	100.0
PGE54614.1	Cry1Ia14	100.0	Cry1Ia2	100.00	Cry1Ia2	100.00	Cry1Ia10	100.0
PGE63619.1	Cry2Ab16	100.0	Cry2Ab3	100.00	*	*	Cry2Ab1	100.0
PGK06240.1	Cry2Aa1	100.0	Cry2Aa9	100.00	*	*	Cry2Aa1	100.0
PGK06243.1	Cry1Ia14	100.0	Cry1Ia2	100.00	Cry1Ia2	100.00	Cry1Ia10	100.0
PGK06244.1	Cry1Aa12	100.0	Cry1Aa3	100.00	Cry1Aa3	100.00	Cry1Aa12	100.0
PGK06250.1	Cry2Ab16	100.0	Cry2Ab3	100.00	*	*	Cry2Ab1	100.0
PGH98789.1	Cry1Ba1	99.9	Cry1Ba5	99.92	Cry1Ba5	100.00	Cry1Ba1	99.9
PGK36565.1	Cry8Aa1	46.1	−	−	Cry8Aa1	47.00	Cry8Aa1	46.1
PGL13502.1	Cry5Ba1	99.6	Cry5Ba1	99.60	*	*	Cry5Ba1	99.6
PGO36223.1	Cry5Aa1	99.7	Cry5Aa1	99.62	*	*	Cry5Aa1	99.7
PGO34936.1	Cry5Ba1	99.8	Cry5Ba1	99.84	*	*	Cry5Ba1	99.8
PGO34728.1	Cry65Aa2	55.6	−	−	*	*	Cry65Aa2	55.6
PGP47008.1	Cry32Wa1	39.0	−	−	Cry41Ab1	34.95	Cry41Ab1	33.7

PGR92655.1	Cry32Wa1	39.2	–	–	Cry41Ab1	35.28	Cry41Ab1	34.0
PGU92045.1	Cry32Wa1	39.0	Cry41Aa1−like	38.02	Cry41Ab1	34.93	Cry41Ab1	33.7
PHD77627.1	Cry2Ab16	100.0	Cry2Ab3	100.00	*	*	Cry2Ab1	100.0
PHD85153.1	Cry2Aa1	100.0	Cry2Aa9	100.00	*	*	Cry2Aa1	100.0
PHD85156.1	Cry1Ia14	100.0	Cry1Ia2	100.00	Cry1Ia2	100.00	Cry1Ia10	100.0
PGY60137.1	Cry32Wa1	39.0	–	–	Cry41Ab1	34.95	Cry41Ab1	33.7
PGW42274.1	Cry14Aa1	44.0	Cry14Aa1	43.98	*	*	Cry14Aa1	44.0
PGW46196.1	Cry14Ab1	42.4	Cry14Aa1	42.46	*	*	Cry14Aa1	42.4
PGW46198.1	Cry14Aa1	41.1	Cry14Aa1	41.19	*	*	Cry14Aa1	41.1
PGY63215.1	Cry21Ba1	28.9	–	–	*	*	Cry21Ba1	28.9
PGY64190.1	Cry70Ba1	97.3	–	–	*	*	Cry31Aa6−like	65.1
PGY64183.1	Cry68Aa1	80.1	–	–	Cry9Aa2	35.88	Cry9Aa1	34.0
PGY64101.1	Cry40Da1	66.9	–	–	Cry40Da1	67.32	Cry40Da1	66.9
PGY63227.1	Cry71Aa1	65.9	–	–	Cry53Ab1	40.44	Cry53Ab1	40.0
PGY73908.1	Cry69Ab1	58.0	–	–	Cry53Ab1	35.63	Cry43Ba1	41.1
PHG40087.1	Cry2Ab16	100.0	Cry2Ab3	100.00	*	*	Cry2Ab1	100.0
PHG55433.1	Cry1Ia14	100.0	Cry1Ia2	100.00	Cry1Ia2	100.00	Cry1Ia10	100.0
PHG55436.1	Cry2Aa1	100.0	Cry2Aa9	100.00	*	*	Cry2Aa1	100.0
PHF50510.1	Cry2Aa1	100.0	Cry2Aa9	100.00	*	*	Cry2Aa1	100.0
PHF50513.1	Cry1Ia14	100.0	Cry1Ia2	100.00	Cry1Ia2	100.00	Cry1Ia10	100.0
PHF69249.1	Cry2Ab16	100.0	Cry2Ab3	100.00	*	*	Cry2Ab1	100.0
PNK22955.1	Cry8Db1	51.1	Cry8Db1	51.12	Cry8Fa1	47.56	Cry8Db1	51.1
PNK22471.1	Cry8Ga2	100.0	Cry8Ga3	100.00	Cry8Ga3	100.00	Cry8Ga2	100.0
PNK25039.1	Cry8Fa1	100.0	Cry8Fa1	100.00	Cry8Fa1	100.00	Cry8Fa1	100.0
PNK32300.1	Cry8Ab1	80.0	–	–	Cry8Ab1	80.27	Cry8Ab1	80.0
PNK28167.1	Cry8Ea3	100.0	Cry8Ea1	100.00	Cry8Ea1	100.00	Cry8Ea1	100.0
PNK28168.1	Cry8Ca1	99.8	Cry8Ca1	99.83	Cry8Ca4	99.83	Cry8Ca1	99.8
PNK22276.1	Cry2Aa1	100.0	Cry2Aa9	100.00	*	*	Cry2Aa1	100.0
PNK41404.1	Cry1Ka2	100.0	Cry1Ka1	99.16	Cry1Ka1	98.94	Cry1Ka1	98.4
PNK40066.1	Cry1Fb3	99.9	Cry1Fb4	99.78	Cry1Fb4	99.82	Cry1Fb3	99.9
PNK40029.1	Cry1Da3	99.9	Cry1Da2	98.69	Cry1Da2	99.82	Cry1Da1	98.7
PNK40024.1	Cry1Ib11	99.9	Cry1Ib9	98.75	Cry1Ib6	100.00	Cry1Ib10	98.7
PNK40012.1	Cry1Ab18	100.0	Cry1Ab18	100.00	Cry1Ab18	100.00	Cry1Ab18	100.0
PNK46490.1	Cry1Bb1	99.9	Cry1Bb1	99.91	Cry1Bc1	100.00	Cry1Bb1	99.9
PNK46492.1	Cry1Hb1	100.0	Cry1Hb1	100.00	Cry1Hb1	100.00	Cry1Hb1	100.0
PNK42250.1	Cry1Id1	100.0	Cry1Id1	100.00	Cry1Id1	100.00	Cry1Id1	100.0
PNK42253.1	Cry1Ja2	100.0	Cry1Ja1	99.57	Cry1Ja1	99.09	Cry1Ja1	99.6
PNK42258.1	Cry1Nb1	100.0	–	–	Cry1Ca4	52.73	PesticidalProtein	100.0
PNK47117.1	Cry8Db1	51.1	–	–	Cry8Fa1	47.56	Cry8Db1	51.1
PNK46110.1	Cry8Ga2	100.0	Cry8Ga3	100.00	Cry8Ga3	100.00	Cry8Ga2	100.0
PNK42405.1	Cry1Ca8	100.0	Cry1Ca9	100.00	Cry1Ca9	100.00	Cry1Ca7	100.0
PNK42101.1	Cry2Ab16	100.0	Cry2Ab3	100.00	*	*	Cry2Ab1	100.0
PNK41517.1	Cry1Ia14	100.0	Cry1Ia2	100.00	Cry1Ia2	100.00	Cry1Ia10	100.0
PNK41087.1	Cry9Ea1	100.0	Cry9Ea2	100.00	Cry9Ea2	100.00	Cry9Ea1	100.0
PNK40022.1	Cry1Da1	100.0	Cry1Da2	100.00	Cry1Da2	100.00	Cry1Da1	100.0
PQQ47595.1	Cry1Ie5	79.3	Cry1Ib4	79.69	Cry1Ib1	81.69	Cry1Ib4	79.7
PQQ47537.1	Cry73Aa1	40.0	–	–	*	*	Cry41Aa1−like	40.0
PTN43520.1	Cry1Ac10	100.0	Cry1Ac9	100.00	Cry1Ac9	100.00	Cry1Ac1	100.0
RBN53067.1	Cry1Ca8	100.0	Cry1Ca9	100.00	Cry1Ca9	100.00	Cry1Ca7	100.0
RBN52662.1	Cry1Ia14	100.0	Cry1Ia2	100.00	Cry1Ia2	100.00	Cry1Ia10	100.0
RBN52569.1	Cry9Ea1	100.0	Cry9Ea2	100.00	Cry9Ea2	100.00	Cry9Ea1	100.0
RBN52453.1	Cry1Da1	100.0	Cry1Da2	100.00	Cry1Da2	100.00	Cry1Da1	100.0
RBN53338.1	Cry2Ab16	100.0	Cry2Ab3	100.00	*	*	Cry2Ab1	100.0
AXY11181.1	Cry41Ca1	100.0	Cry41Ba2	63.87	Cry41Ba2	72.64	Cry41Ba2	63.5
AXY11292.1	Cry32Wa1	100.0	Cry41Aa1−like	57.91	Cry32Da1	40.24	Cry41Aa1−like	58.2
AXY11305.1	Cry32Va1	100.0	Cry32Aa1	58.09	Cry32Aa1	41.34	Cry32Aa1	56.3
AXY11484.1	Cry32Ma1	57.7	Cry32Aa1	54.52	Cry41Ab1	52.93	Cry32Aa1	53.4
AYF84829.1	Cry8Fa1	100.0	Cry8Fa1	100.00	Cry8Fa1	100.00	Cry8Fa1	100.0

AYF84849.1	Cry8Ea3	100.0	Cry8Ea1	100.00	Cry8Ea1	100.00	Cry8Ea1	100.0
AYF84850.1	Cry8Ca1	99.8	Cry8Ca1	99.83	Cry8Ca4	99.83	Cry8Ca1	99.8
AYF84876.1	Cry8Ab1	80.0	–	–	Cry8Ab1	80.27	Cry8Ab1	80.0
RNG36219.1	Cry3Aa1	100.0	Cry3Aa7	100.00	Cry3Aa7	100.00	Cry3Aa1	100.0
AZR80780.1	Cry21Ca2	64.6	Cry21Ba1	50.22	*	*	Cry21Aa2	48.1
AZR80678.1	Cry5Ba1	27.2	Cry5Ba1	27.62	*	*	Cry5Ba1	27.2
AZR80706.1	Cry21Aa2	99.7	Cry21Aa2	99.66	*	*	Cry21Aa2	99.7
AZR80772.1	Cry21Aa2	44.6	Cry21Aa2	46.34	*	*	Cry21Aa2	44.6
AZR80774.1	Cry21Ca2	65.9	Cry21Ba1	50.08	*	*	Cry21Ba1	50.0
RVU62660.1	Cry12Aa1	100.0	Cry12Aa1	100.00	*	*	Cry12Aa1	100.0
RYS59630.1	Cry2Aa1	100.0	Cry2Aa9	100.00	*	*	Cry2Aa1	100.0
RYS59633.1	Cry1Ia14	100.0	Cry1Ia2	100.00	Cry1Ia2	100.00	Cry1Ia10	100.0
RYS58618.1	Cry2Ab16	100.0	Cry2Ab3	100.00	*	*	Cry2Ab1	100.0
QBP15688.1	Cry11Aa1	100.0	Cry11Aa3	100.00	*	*	Cry11Aa1	100.0
QBP15695.1	Cry4Ba1	100.0	Cry4Ba5	100.00	cry4Ba	100.00	Cry4Ba	100.0
QBP15696.1	Cry10Aa3	100.0	Cry10Aa4	100.00	Cry10Aa4	100.00	Cry10Aa3	100.0
QBP15732.1	Cry4Aa2	100.0	Cry4Aa4	100.00	Cry4Aa4	100.00	Cry4Aa2	100.0
TBX61382.1	Cry9Ea1	100.0	Cry9Ea2	100.00	Cry9Ea2	100.00	Cry9Ea1	100.0
TBX73091.1	Cry1Ab12	100.0	Cry1Ab9	100.00	Cry1Ab14	100.00	Cry1Ab1	100.0
TBX72000.1	Cry1Aa11	100.0	Cry1Aa19	100.00	Cry1Aa5	100.00	Cry1Aa1	100.0
TBX99229.1	Cry1Ia14	100.0	Cry1Ia2	100.00	Cry1Ia2	100.00	Cry1Ia10	100.0
TBX99235.1	Cry1Ca8	100.0	Cry1Ca9	100.00	Cry1Ca9	100.00	Cry1Ca7	100.0
TBX99239.1	Cry1Da1	100.0	Cry1Da2	100.00	Cry1Da2	100.00	Cry1Da1	100.0
TBX88898.1	Cry2Ab16	100.0	Cry2Ab3	100.00	*	*	Cry2Ab1	100.0
QBM59570.1	Cry11Aa1	100.0	Cry11Aa3	100.00	*	*	Cry11Aa1	100.0
QBM59577.1	Cry4Ba1	100.0	Cry4Ba5	100.00	cry4Ba	100.00	Cry4Ba	100.0
QBM59578.1	Cry10Aa3	100.0	Cry10Aa4	100.00	Cry10Aa4	100.00	Cry10Aa3	100.0
QBM59613.1	Cry4Aa2	100.0	Cry4Aa4	100.00	Cry4Aa4	100.00	Cry4Aa2	100.0
TEA80813.1	Cry5Ba1	48.7	Cry5Ba1	48.81	*	*	Cry5Ba1	48.7

* BtToxin_scanner web−server was unavailable when benchmarking was performed, thus, we provide data for comparison between the older version of Cry processor, proteins that were neglected by the older version of CryProcessor are marked with asterisks (github hash 4b0dc).
